# “It’s what we perceive as different”: an interpretative phenomenological analysis of Nigerian women’s characterization of their health during the COVID-19 pandemic

**DOI:** 10.1186/s12905-024-03259-w

**Published:** 2024-07-18

**Authors:** Mary Ndu, Gail Teachman, Janet Martin, Elysee Nouvet

**Affiliations:** 1 Department of Health and Rehabilitation Sciences, Faculty of Health Sciences, Western University, 1151 Richmond Street London, Ontario, N6A 3K7 Canada; 2 School of Occupational Therapy, Faculty of Health Sciences, Western University, 1151 Richmond Street London, Ontario, N6A 3K7 Canada; 3 Centre for Medical Evidence, Decision Integrity, Clinical Impact (MEDICI), Schulich School of Medicine and Dentistry, Western University, 1151 Richmond Street London, Ontario, N6A 3K7 Canada; 4 Faculty of Health Science, Western University, 1151 Richmond Street London, Ontario, N6A 3K7 Canada

**Keywords:** COVID-19, Interpretive phenomenological analysis, Nigeria, Global health, Social determinant of health, Lived experiences

## Abstract

**Background:**

Health has historically been adversely affected by social, economic, and political pandemics. In parallel with the spread of diseases, so do the risks of comorbidity and death associated with their consequences. As a result of the current pandemic, shifting resources and services in resource-poor settings without adequate preparation has intensified negative consequences, which global service interruptions have exacerbated. Pregnant women are especially vulnerable during infectious disease outbreaks, and the current pandemic has significantly impacted them.

**Methods:**

This study used an interpretive phenomenological analysis study with a feminist lens to investigate how women obtained healthcare in Ebonyi, Ogun, and Sokoto states Nigeria during the COVID-19 pandemic. We specifically investigated whether the epidemic influenced women’s decisions to seek or avoid healthcare and whether their experiences differed from those outside of it.

**Results:**

We identified three superordinate themes: (1) the adoption of new personal health behaviour in response to the pandemic; (2) the pandemic as a temporal equalizer for marginalized individuals; (3) the impacts of the COVID-19 pandemic on maternal health care. In Nigeria, pregnant women were affected in a variety of ways by the COVID-19 epidemic. Women, particularly those socially identified as disabled, had to cross norms of disadvantage and discrimination to seek healthcare because of the pandemic’s impact on prescribed healthcare practices, the healthcare system, and the everyday landscapes defined by norms of disadvantage and discrimination.

**Conclusion:**

It is clear from the current pandemic that stakeholders must begin to strategize and develop plans to limit the effects of future pandemics on maternal healthcare, particularly for low-income women.

**Supplementary Information:**

The online version contains supplementary material available at 10.1186/s12905-024-03259-w.

## Introduction

Throughout history, pandemics have had an adverse effect on the social, economic, and political landscape of health. The increase in new pathogens and the resurgence of old pathogens continue to be a global concern as the current pandemic reduces its hold on the world [1–4]. As pathogens multiply, so do risks of mortality and comorbidity from its consequences [2–4]. The current pandemic has reinforced the adverse impact of redistributing resources and services in resource-poor settings without adequate preparedness, exacerbated further by service disruptions worldwide [1, 5–8]. We know that pregnant women are particularly vulnerable during infectious disease outbreaks, and it has been shown that the current pandemic has had a significant impact on pregnant women globally [5, 7–11]. The pandemic has the potential to reverse years of progress made in women’s health and threatens to hinder the achievement of Sustainable Development Goal (SDG) three (3), which aims to achieve universal health coverage, including ensuring that people should have access to safe, adequate, quality, and affordable essential medicines and vaccines and financial risk protection [12, 13].

Previous experiences from the Ebola epidemic in West Africa have shown that access to healthcare services is disrupted during public health crises [14–22]. The fragility of health systems and emergency preparedness have been tested during the COVID-19 pandemic, which poses a great danger for Nigeria, where access and utilization of maternal, newborn, and child health services [10, 23–32] and utilization of maternal, newborn, and child health services are still poor [10, 23–32]. The COVID-19 pandemic is believed to have impacted Nigeria’s utilization of maternal and newborn child health services [31, 33]. However, few studies have formally evaluated the extent, direction, contextual factors, and perceptions of patients regarding these changes in the country. Furthermore, the effects of the lockdown and movement restrictions imposed by the government to control community spread on maternal and child health in Nigeria have been largely unexplored.

This article draws on data collected for an interpretive phenomenological analysis (IPA) study using a feminist lens that explored how women accessed healthcare in Ebonyi, Ogun, and Sokoto states of Nigeria during the COVID-19 pandemic. As the methodological framework for this study, IPA is specifically relevant in exploring the complex experiences of women as mothers with children. The methodology is designed for researchers to engage deeply with the subjective experiences of research participants [34, 35]. The aim is to offer a nuanced understanding of participants’ experiences when studying personal and emotional responses to an issue. We found it suited our study as it provided a framework for an empathetic and detailed exploration of women’s lived experiences, perceptions, opinions, thoughts, and feelings during the COVID-19 pandemic. Our rationale for employing IPA comes from its theoretical foundation in phenomenology, idiographics, and hermeneutics. IPA does not only seek to elucidate factual experiences, it interprets how individuals make sense of their world [36, 37]. In the context of our study, it means highlighting the nuances of women’s daily lives and the challenges that they faced during the COVID-19 pandemic. These experiences are often shaped by unique sociocultural factors encompass the adaptations, resilience, and struggles of being a mother during the COVID-19 pandemic. IPA’s focus on exploring such nuanced of personal experiences aligned with our study objective, which aimed to reveal the layered experiences of women during the COVID-19 pandemic. The qualitative study’s primary objective was to gain an in-depth understanding of women’s encounters with healthcare and their perspectives on seeking care during the peak of the pandemic. Specifically, we investigated whether the pandemic influenced women’s decisions to seek or avoid healthcare and whether their experiences of seeking healthcare during the pandemic differed from those outside the pandemic. It is important to note that the intention of this study was not to generalize women’s experiences, but rather to contribute to health equity, pandemic preparedness, and response strategies, and generate evidence that can inform national health policies with an emphasis on the gendered impacts of public health emergencies in Nigeria and beyond.

### Conceptual framework

The conceptual framework of this study is based on the World Health Organization’s (WHO) concept of responsiveness. The WHO defines health system responsiveness as the capability of the health system to fulfill the legitimate expectations of the population regarding their engagement with the health system, aside from the expectations for improvements in health or wealth [38]. The framework outlines that the population’s expectations should be guided by international human rights norms and professional ethics, which posits that all humans have a fundamental right to access essential services whenever the need arises. The emphasis on responsiveness underscores the importance of designing health systems based on the needs of the end-users [39–42] and aligning them with the broader health system objectives of ensuring equitable access to healthcare services for the entire population. Our framework builds on the existing health systems responsiveness framework proposed by Mirzoev & Kane, (2017). Using the WHO framework, the authors extend the framework by proposing a comprehensive conceptual framework for health systems responsiveness that places the experiences of interaction at the core of the health system and recognizes the determinants of responsiveness from the systems and people’s perspectives. Mirzoev and Kane (2017) suggest that by understanding the interaction between the health system and the user, the system’s responsiveness can be improved. They also propose a framework that considers the various factors that influence responsiveness, such as the availability of resources, the quality of service, and the attitudes and behaviours of both the health system and the user.

**Fig. 1:**
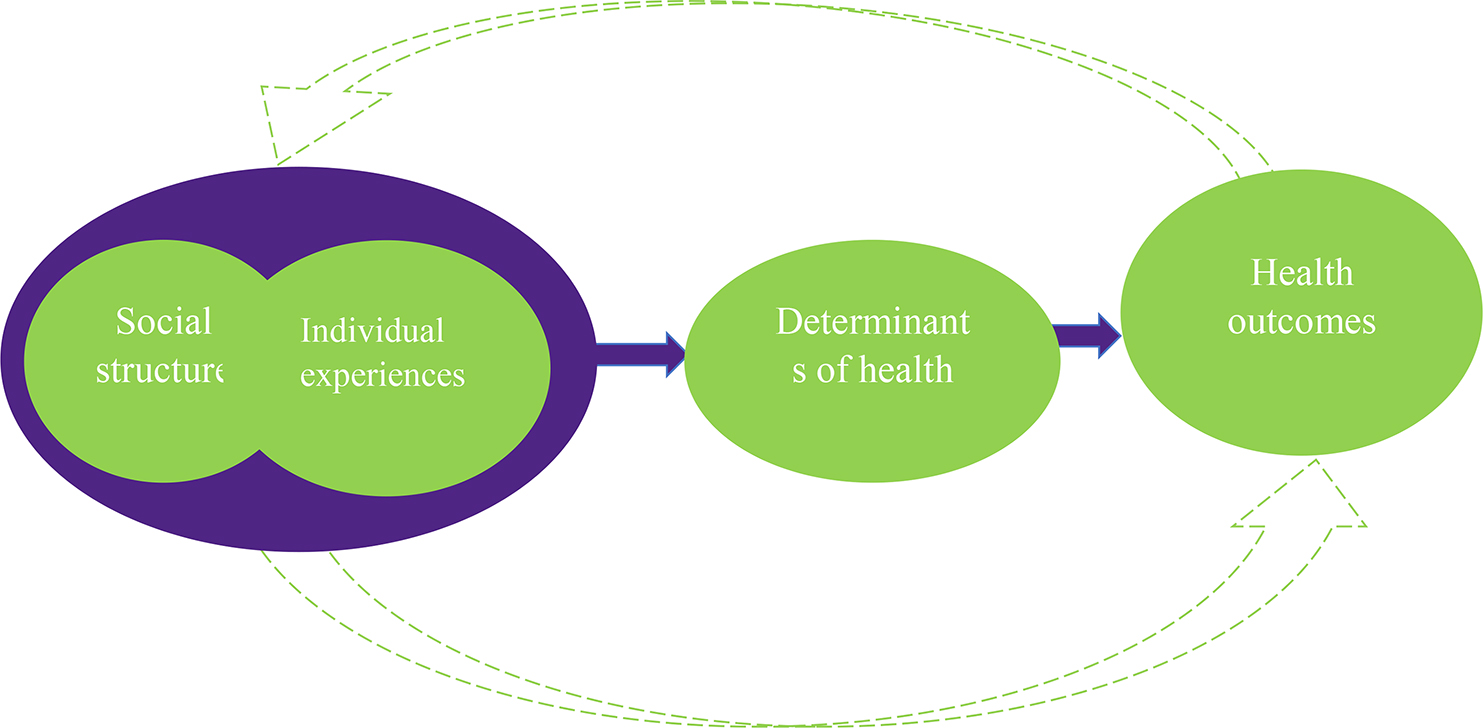
Conceptual framework of responsiveness adapted from Mirzoe & Kane, (2017)

We know that how women make decisions about their health-seeking during pregnancy, delivery, and postpartum has significant implications for maternal and child health outcomes. As such, we acknowledge that the interaction of women with systems shapes their experiences and the decisions that make about their health care. However, what we propose, which in our opinion is rarely examined, is the inclusion of changing social dynamics and the role of health systems in amplifying unheard voices. See Fig. 1. We go further in our conceptualization to recognize the role the dynamic of change and historically relevant factors such as coloniality and coloniality of gender continue to play in shaping social structures in low- and middle-income countries like Nigeria, which has implications for understanding the ways determinants of health such as norms and cultural experiences are shaping women’s health-seeking behaviour. This is especially important as these social structures, norms, and cultural experiences are embedded in the experiences of inequality that are deeply rooted in colonial and post-colonial histories, which have shaped the current health landscape of Nigeria and its women.

## Method

The research methodology adopted for this study was Interpretative Phenomenological Analysis (IPA), which was combined with a feminist perspective to scrutinize, to the extent feasible, the perceptions and experiences of women in Nigeria. The interpretive phenomenological approach in analyzing and interpreting different aspects of the pandemic experience allows us to gain a deeper understanding of how individuals experience and make sense of the world around them, particularly in the context of a major global event such as the COVID-19 pandemic. Nonetheless, it is pertinent to note that this approach requires the researcher’s interpretive engagement, often referred to as the use of double hermeneutics [43–49]. Consequently, the study presents detailed interpretative accounts for a small group of participants rather than a generalized overview of larger sample size, in adherence to the tenets of IPA [47].

### Study design and setting

This study utilized the Interpretative Phenomenological Analysis approach to explore women’s experiences with the health system in Nigeria during the pandemic. This research method examines their lifeworlds and the meanings they derive from their experiences. Moreover, IPA gives researchers a flexible, iterative analytical process where data collection and analysis are tightly interwoven, ensuring the findings are grounded in study participants’ perspectives [44, 47]. This approach is valuable because it respects participants’ individuality. In the context of our study, this means each mother’s experience is analyzed and interpreted within its own merit, avoiding broad generalizations. Instead, we highlighted the particularities and complexities of each women’s story. As such, using IPA provided a space for silent voices that are often underrepresented or subsumed within generalization in research.

Participant selection is a critical step in IPA, which, like other phenomenological studies, uses a purposive sampling technique to aim for participants that will provide rich and robust data. We purposively selected mothers who could provide rich and detailed narratives about their experiences during the COVID-19 pandemic. As such, our specific selection criteria focused on recruiting women who represent diverse maternal experiences, including different socioeconomic backgrounds, ages, and numbers of children. This diversity in participants is vital in conducting IPA studies as participants perceive and make sense of their personal and shared experiences differently.

Following the IPA format, we designed in-depth semi-structured interviews using open-ended questions to encourage women to speak freely about their perceptions, experiences, thoughts, and opinions of the COVID-19 pandemic and its implications for them and their children’s health. The questions were designed to prompt the women to reflect and elaborate on their responses and delve into the emotional aspects of mothering and being a woman during the COVID-19 pandemic. Using this interview guide, we answered the research question, “Do women of childbearing age characterize their health-seeking experiences for themselves or their children during the COVID-19 pandemic as different than health-seeking outside the pandemic? Why or why not?” When compared to other forms of qualitative inquiry, IPA requires an in-depth analysis of participants’ individual experiences, as such, the recommended sample size in IPA is 3–15 to allow the researcher to delve deep into the analysis. However, given our criteria for diversity, we recruited a larger sample size for whom the research question was meaningful. A large sample size for a global event like the COVID-19 pandemic provided a rich collection of stories from which we could pool robust and meaningful responses to answer the research question. While IPA does not aim to generalize experiences, it looks for divergence and convergence that could explain why participants experience similar realities differently.

The study’s analytical process was guided by a detailed and systematic analysis of interview transcripts. The initial process begins with researchers reading each transcript multiple times to gain a sense of the whole before delving into a more detailed and interpretive phase. Immersing in the data allows the researcher to examine and note the unique expressions, words, or phrases participants used in the interview. In our analysis, using this approach involved noting the initial thoughts of participants through their responses, developing the emergent themes, and searching for any connections between the themes across our three cases. The process involved multiple readings and coding of the data using an iterative process. This process not only emphasized each woman’s unique experiences but also allowed a convergence pattern to form across the stories in line with the idiographic focus of IPA while acknowledging the women’s shared experience of the same phenomena. This approach ensured that interpretation remained as close to the women’s description of their experiences as possible without losing the essence of their experiences. Interpretation here refers to the work that the researcher(s) undertake(s) to understand and represent the participants’ claims, stories, feelings, and concerns. Here, we draw on participants’ accounts to make generalizable claims, doing so with caution to acknowledge the limitations of such generalizations as we embed them in the context of the study. In recognition of this limitation, IPA interpretation is co-constructed between the researcher and the participants. As such, in our interpretation, we did not speculate. Instead, we presented the descriptions and stories of the women in the context of the study. The co-construction process in our study began with the interview, where we used our analytical skills, perceptions and insights in the interview. At the same time, the women contributed by sharing their personal experiences and the meaning they ascribed to these experiences. During the interview process, we constantly reflected on our reactions to responses and how they could influence the interview and data analysis process. We documented observations and insights in the field notes at the end of every interview. This critical reflexive approach toward data construction and analysis invites readers to recognize how researchers may have influenced the study and its findings.

The study was set in three states selected from three geopolitical zones of Nigeria, Southwest (Ogun), Southeast (Ebonyi), and Northwest (Sokoto), each with distinct sociocultural identities. The groups hold strong cultural and personal belief systems about health and health care, which have been shown to influence their health-seeking behaviour and general social wellness. To capture the complexity of Nigeria’s sociocultural and socioeconomic dynamics, we focused on a geographically diverse selection of states that provides a robust representation of this complexity. Each of the states selected encompasses a diverse population in the socioeconomic quadrant. For instance, Ebonyi State, a young state compared to Ogun and Sokoto, provides an opportunity to understand the challenges of development and health in addressing the health issues in hard-to-reach areas. The state also represents eastern Nigeria’s socioeconomic characteristics, with small-scale farming and trading as the main economic activity. It also provides insight into the cultural nuances of the Igbos in eastern Nigeria. Ogun state, which borders Lagos state—an African economic hub, has developed as a significant player within Nigeria’s economy, attracting a diverse pool of Nigerians. The state population is a rich mix of urban and semi-urban population with different socioeconomic status. The state provides insights into the implications of rapid urbanization on health-seeking behaviour. It also provides insights into the dynamism of the Yoruba people, one of the oldest states in the western region with a growing health system. Sokoto state, which is predominantly rural and has a cultural base in northern Nigeria, provides insight into the developmental challenges and impact of religion on health-seeking. Each of these embodies a unique socioeconomic pattern that represents their region. The decision to focus on these three regions was partly based on resource limitations, making it difficult to conduct the study with a broader scope and the lead researcher’s networks in these regions through our local NGO.

### Sampling

Phenomenology-based research typically adopts a criterion sampling approach, a variation of purposive sampling that involves identifying participants who meet predefined criteria [50, 51]. One of the most prominent criteria within this approach is the participant’s direct experience with the phenomenon under investigation. Researchers seek out participants who have experienced the phenomenon (in this study that is healthcare seeking or perceived potential need for healthcare during COVID-19), but who may differ in personal characteristics and their individual experiences of the phenomenon. This approach allows for a more comprehensive and nuanced understanding of the phenomenon by generating a diverse range of perspectives and experiences. The study aimed to conduct 24 in-depth interviews (IDIs) in all three states of Nigeria, with eight women interviewed in each state. A subset of eight women was selected based on a defined post-hoc selection criteria for this paper because of the themes that arose during analysis specifically for those women accessing hospitals. The women selected for the study lived within rural (4) and semi-urban communities (4), identified as married, were either pregnant, delivered, or at least had one under-five child during the pandemic restrictions. Additional criteria for inclusion required the women to be aged 15–49 years old, must have visited a hospital for any service during the pandemic, or had a need to visit a hospital and chose not to do so. Although the study sampled women who needed health care and did not visit a hospital, in this paper those women are excluded to highlight the experiences of accessing care during an environmental disruption. Table 1 below provides demographic information.

**Table 1:** Participant demographic information

Participants demographics		Number
Age, years	15–30	14
	31–45	9
	36–49	1
Education		
	Secondary school	13
	Bachelor	6
	College i.e., HND	5
Employment Status		
	Paid employment	6
	Self employed	10
	Unpaid employment	8
Population area		
	Semi-Urban	10
	Rural	14

### Data generation

Following the IPA format, we designed in-depth semi-structured interviews using open-ended questions to encourage women to speak freely about their perceptions, experiences, thoughts, and opinions of the COVID-19 pandemic and its implications for them and their children’s health. The interview questions were designed to prompt the women to reflect and elaborate on their responses and delve into the emotional aspects of mothering and being a woman during the COVID-19 pandemic. To recruit the women, the research team contacted women groups in the selected locations for an introductory meeting with groups of potentially interested women to inform them of the study. From these introductory meetings, women who indicated interest in participating and met our selection criteria were invited to participate in a focus group discussion. At the end of the focus group discussion, the research identified women who were determined to have interesting stories that required further exploration.

Before the focus group discussion, the research team obtained written informed consent to participate in the study with a second verbal informed consent to participate in the interview provided during the interview. The research assistants subsequently contacted the, women, scheduled, and confirmed the dates and times of the planned interviews. Where a teenage mother was involved, a caregiver, i.e., mother-in-law or partner, provided informed written and verbal consent. Before the study commenced the tools were translated into three Nigerian languages commonly used in the states—Igbo, Hausa, and Yoruba. Translations was done through the Nigerian Translators Association to ensure it is linguistically accurate.

The interviews lasted approximately 30 to 60 min and were conducted in English, Igbo, Hausa, and Yoruba (see attachment 1). The primary data collection team consisted of speakers of the three languages and were trained by a local research NGO in conducting data collection using local languages. To ensure an appropriate environment for the one-to-one interviews, a separate room was selected, free from external distractions or interruptions, to facilitate an uninterrupted exchange between the interviewer and interviewee. The interviews were recorded for transcription and documentation purposes, and strict confidentiality was maintained throughout the research process. In the event of emotional distress or discomfort experienced by the women during the interview, adequate psychological support was provided to prevent secondary psychological harm. After the interview, the research assistants transcribed the script back to English which was then verified by an expert. The interview guides captured barriers and facilitators that influenced women’s health decision-making during COVID-19 as well as their overall experiences engaging with health care in the period under study. To facilitate neutral and objective data collection, the research team maintained an impartial stance and established good relationships with the women. The lead researcher employed active listening and clarification techniques to promote authenticity in the data and to prevent any potential bias.

### Data analysis

This paper is based on the analysis of seven in-depth interviews selected from 24 interviews in the main study purposively selected for their specific experiences accessing health care during the period. The analytical process comprised four distinct steps: firstly, a comprehensive reading of all the available material was conducted to form a holistic impression of the data. Next, the researcher identified meaningful units to capture diverse aspects of the women’s experiences, which were subsequently categorized through a coding process. Afterward, the contents of each interview’s coded group were condensed and summarized to capture its essential features. The last step was combining the descriptions and contents of the coded interviews into themes to capture similarities in experiences and perspectives across the seven participants. The methodological approach enabled a thorough and systematic analysis of the data. Using an inductive approach, we read and re-read the interview transcripts to ensure that any new ideas and insights are identified. When each transcript was analyzed and the subthemes identified, the patterns across all the interview transcripts were examined, and superordinate themes were generated that captured the shared experiences of participants. The lead researcher, who led the interview, transcription, and analysis, kept a reflective journal throughout the research process, and during data analysis, the impact of her positionality was taken into account.

### Ethical approval

was obtained from the Research Ethics Board at Western University, Nigeria National Ethics Review Board at the Federal Ministry of Health, and the Ethics Board of Ebonyi, Ogun, and Sokoto States. No identifiers were used during the interviews and focus group discussions while transcribing. The same approach was used to maintain the confidentiality of the participants. Participation in the study was voluntary, and participants knew they could decide to opt-out at any point. Both verbal and written informed consent were obtained from participants.

## Results

In this section, we present three superordinate themes that emerged from the analysis. The themes are: (1) Adoption of new personal health behaviour in response to the pandemic; (2) The pandemic as a temporal equalizer for marginalized individuals; (3) Impacts of the COVID-19 pandemic on maternal health care. The first theme, transformations of personal health behaviour in response to the pandemic is a segue to the following themes as it indicates that women perceived the pandemic as having a positive impact on their own and their family’s health-seeking behaviour. The names of participants have been changed to maintain confidentiality.

Three women delivered without complication. Four women used a government hospital, and three used private primary care. Two women identified as living with a disability.

One of the women reported she had full eclampsia during pregnancy, and the baby died a few weeks later because of organ failure possibly due to exposure to the antihypertensive medication during pregnancy. One woman reported she was diagnosed with COVID-19 and was admitted for a few days before being discharged to home care because of a lack of oxygen in the hospital.

### Theme 1: adopting new personal health behaviour in response to the pandemic

In this theme, three women reflect on new personal health behaviours they perceived are attributable to the pandemic. They describe factors such as adopting new hygiene practices, increased awareness of personal hygiene, and recognition of the benefits of nose covers to show how the pandemic has shaped their new world and made these new behaviours critical components of daily life.

In the below quote, Oba reflects on how the pandemic has influenced her behaviour, noting that she is now more mindful of her actions, particularly regarding hygiene.

*Well*,* I want to say that ever since that COVID time*,* I have been mindful of it [health behaviour]. It has become a part of me. Before I touch anything*,* I am careful to the extent that even at work*,* I wash my hands regularly. People ask me what happened*,* and I tell them that we were taught at work and everywhere that we should not touch things around*,* and thank God*,* we’ve been doing that. [Oba*,* Bank Teller]*

In describing that she has become “mindful”, Oba draws attention to the way that the pandemic has led to increased awareness of the potential risk of infection, which has resulted in changes in behaviour. In becoming more mindful of her actions, she took proactive steps such as washing her hands regularly, avoiding touching things around her, and using face masks. This behaviour change has become a “part of” her daily routine, indicating a new way of being in the world shaped by the pandemic and she perceives it as essential to protect herself and her family from infections.

Oba also used the word “careful” to describe the precautionary measures she adopted to maintain her health and that of her family. Oba’s wording suggests understanding that daily handwashing will minimize the risk of infections. Oba describes her role as a health promoter implying, that she encourages others to adopt good personal hygiene practices and prevent the spread of infections using knowledge acquired from her work.

In the extract below, Ibironke reflects on the change in her health behaviour since the pandemic.

*Yes*,* it has improved [health behaviour]. I noticed that even before that time*,* I usually had infections. So*,* I think it has improved by keeping things away and taking care of my children by washing their hands regularly. Now*,* we are better. [Ibironke*,* Student]*

Ibironke noted that her health and that of her family have improved since the pandemic began, suggesting that they may have been more vulnerable to illness before the pandemic. She used the word “usually” to describe her history of experiencing frequent infections. It suggests that she accepted the frequency of experiencing infections as a normal occurrence before the changes she shared in her health behavior. It also implies that she used the word “usually” to indicate that she is no longer experiencing infections as frequently as before to support her assertion of improved health.

She attributed her improved health to increased hygiene practices, specifically washing hands regularly and keeping things away. She used the phrase “keeping things away” to suggest that she became more health-conscious and became proactive in her methods for protecting herself and her family. She also used the word “regularly” to describe the frequency of handwashing and avoiding touching things around them as a symbol for consistently observing positive behaviour. She used the word “improved” to support her assertion of a shift in behaviour and overall health for herself and her family. Her statements indicate a shift in her daily habits and practices due to the pandemic. This change may be viewed as a new way of experiencing the world that has been brought about by the pandemic.

In the interview extract below, Esther reflects on her continued use of a nose mask even after the COVID-19 pandemic has subsided in her community.

*You see the nose cover*,* now even though there is no COVID*,* I still use my nose mask because I see that it is something good*,* it is not only to contact the virus that is why they said we should be using it I can see that is good for people to be using the nose cover although I don’t use it every day*,* but I do use it*,* and I wash my hands*,* my children too their hygiene I don’t take it lightly. [Esther*,* No paid employment]*

Esther described how she perceived using the nose mask as a good practice, not just for preventing the transmission of the virus, but for overall personal hygiene. This suggests that she has internalized the importance of hygiene practices due to the pandemic. It indicates a shift in her daily habits and practices due to the pandemic. She noted that she has incorporated a nose mask and hand washing into her family’s daily routine and is mindful of their children’s personal hygiene. It suggests that the pandemic has had a lasting impact on her experiences and practices, resulting in a new way of being in the world that is likely to persist even after the pandemic subsides.

Esther used the word “still” to describe her continued use of the nose mask since the pandemic. In her perception, she recognized the benefit of using it, and even though she perceived that there is no longer a COVID-19 pandemic, she still uses nose masks because she sees it as a good practice that can prevent the spread of infections. This suggests that at least some Nigerian women came to recognize through their experience with COVID-19 the importance of nose masks as a preventative measure against infections.

The above interview extracts demonstrate that the COVID-19 pandemic has resulted in significant personal and social transformations, particularly among women. The profound changes women experienced because of the COVID-19 pandemic fundamentally altered health-seeking behaviours, highlighting a shift towards a more hygiene-consciousness. The stories from Oba, Ibironke, and Esther, provided a vivid illustration of this transformation. It also offers insight into how environmental disruptions such as the COVID-19 pandemic can induce new health behaviours that can be imbibed into existing social norms. For example, Oba’s story illustrated how an increased awareness of hygiene initially nudged by COVID-19 protocols, evolved into a sustained proactive practice in her home and workplace. Her initiative to take a proactive approach to regular handwashing and cautious interactions with herself and her family, indicated her willingness to change her behaviour and adopt a healthier alternative. This simple shift in behaviour which otherwise might have been temporary, became a permanent lifestyle change for her and her family. Oba’s story showed that community members can own their health and champion a health promotion campaign with adequate information. She provided evidence that policymakers can capitalize on to incorporate and support hygiene education in non-traditional health settings i.e. workplace to shift health behaviour. Similarly, Ibironke’s story provided evidence of the direct benefit of increased hygiene practices on health outcomes. Her proactive measures like hand washing and minimizing contact with potentially contaminated surfaces, protected her and her family. Ibironke’s story further emphasized the need for simple, consistent health promotion programs in communities. Like Oba and Ibironke, Esther’s personal decision to continue to use masks after the COVID-19 pandemic subsided, highlighted a significant shift in public perceptions of public health preventive measures However, such health policies should incorporate and support continuous health promotion initiatives that take advantage of these new shifts in behaviour as an integral part of the broader public health strategy.

### Theme 2: the pandemic as a temporal equalizer for marginalized individuals

In this theme, we explore the positive and negative impacts of the pandemic on women living with disabilities. The below interview extracts are from two women who self-identify as living with a disability. When describing their experiences during the pandemic, these women focused on the positive effects of the pandemic, describing how specific changes provoked by the pandemic provided them with a reprieve from the discrimination and stigma they usually face in healthcare settings.

For instance, Bukky described her usual behaviour of staying indoors and keeping to herself before the pandemic. She describes her norm being to only leave home when called for catering services by customers, or antenatal appointments.

*I don’t usually go out. I only went out for my antenatal appointments during my pregnancy. I usually keep to myself and even lock up my shop. However*,* if any of my customers call me for catering services*,* then I go out. [Bukky*,* Caterer]*

Bukky’s norm, connected to her disability, is to seek privacy and avoid experiencing discomfort in social situations. Her willingness to leave home only for work outside the pandemic may indicate a need to maintain income and/or commitment to her catering business and customers. The significance of Bukky’s account of her usual way of being emerged in her description of her health experience during COVID-19, and its impact on her experience accessing healthcare services.

Before the pandemic, Bukky described how people treated her, often avoiding her in public. She described how her disability often made people avoid her in healthcare settings. However, during the pandemic, she explained that she could go to the hospital and see a healthcare provider without worrying about people avoiding her. In her description, she notes that COVID-19 provided some relief from dealing with people staring at or avoiding her.

*You know that*,* that COVID-19 period*,* they always say we should stay in our house*,* so as for me*,* the little disability or should I call it a disability*,* that I have made people run away somehow*,* but when the COVID-19 came*,* it pays me a little because when I went to the hospital*,* nobody will be looking at me anyhow nobody will do anyhow*,* I will go*,* and I will see the nurse or doctor*,* and they will treat me*,* and I will come back. [Bukky*,* Caterer]*

Bukky’s experience highlighted the positive impact of the pandemic on her access to healthcare services. She used the phrase “staying at home” to describe the restrictions on movement during the pandemic to prevent the spread of the virus. She perceived this as having some positive effects on her health-seeking behaviour. Her use of the phrase “pays me a little” indicates an acknowledgment of the pandemic’s negative and positive effects on her daily life. When Bukky used the phrase “little disability,” it indicates a struggle to define her disability and the possibility of her questioning whether to refer to her condition as a disability. The word “little” minimizes her disability and indicates a need to project an able-bodiedness based on social standards and a struggle to understand the social rejection. She emphasized the positive aspect of the pandemic while using the words “looking” and “anyhow” to describe her negative experience and her perception of discrimination and stigma when trying to access care before the pandemic because of her disability. She attributes the positive change in experience and better treatment from healthcare providers to COVID-19.

Similarly, Halima, who also identifies as a person living with a disability, shared her experience of how the COVID-19 pandemic affected her access to antenatal care. She describes that during her first pregnancy, she received poor treatment due to her disability. She described how a nurse discouraged her from coming for antenatal care because this would involve being around people and they would stare at her. However, during the pandemic, she could attend her antenatal care visits regularly because there were fewer people in the healthcare facilities, and she received better treatment.

*During my first pregnancy*,* I didn’t go for ante-natal often. Even there was a time when the nurse called me that I should not come because of the way people were looking at me. She said that I should not come often*,* but during the lockdown*,* I went to the hospital very well for my second pregnancy because there were not many people. [Halima*,* No paid employment]*

Halima speaks of “not coming often” as she qualifies the infrequency of her antenatal visits prior to the pandemic and based at least in part on a nurse’s advice. Similar to Bukky, Halima perceived the looks of people as a form of discrimination and stigma.

Bukky and Halima’s perception indicates that the COVID-19 pandemic may harbor some positive impacts on women with disabilities. Both women perceived the pandemic as providing them with a reprieve from the discrimination and stigma they usually face in healthcare settings. Bukky mentions that during the pandemic, healthcare workers treated them without discrimination, and they received the necessary medical attention. This is a significant improvement from her experience before the pandemic, where she felt people avoided her due to their disability. Similarly, Halima mentions that during her first pregnancy, she received poor treatment due to their disability, and healthcare workers discouraged her from coming for antenatal care. However, during the pandemic, they could attend their antenatal care regularly because there were fewer people in healthcare facilities, and they received better treatment.

The experiences of Bukky and Halima during the COVID-19 pandemic offer valuable lessons on the positive possibilities of an inclusive healthcare system. Suppose policymaker continues to foster the conditions that reduced discrimination and increased access to healthcare during the COVID-19 pandemic. In that case, the health system can progress significantly towards a more equitable healthcare system for differently-abled persons. Bukky’s and Halima’s stories represent the voices of members of the Nigerian society who while differently abled must continue to exist within a society where ableism is defined from the lens of othering. To address this view will require a multisectoral collaborative effort, involving differently-abled persons, governments, health providers, and civil society groups. Policymakers must ensure that health policies specifically address the needs of differently-abled people, recognizing the unique challenges they experience in their daily lives accessing social and health services. Nigeria’s health system still has much to do to be inclusive and equitable, especially for this population. Therefore, exploring measures to mitigate these challenges affecting their access to quality care is essential. In addition to developing and strengthening health policies, there is a need to re-educate primary health providers on customer-related skills when providing care to differently-abled people. This training must address ableism in healthcare settings similar to Nigeria’s health system. The stigma and skewed perceptions some health providers hold determine the kind of care and the treatment they provide to differently-abled persons. It will not only enhance their quality of life but enrich the healthcare system, making it more responsive and sensitive to the needs of all its users.

### Theme 3: impacts of the COVID-19 pandemic on maternal health care during the COVID-19 pandemic

In this theme, we present the stories of lived experiences of some women who delivered during the pandemic. Their stories raise awareness of the unique challenges that women faced during the pandemic and validation for other women who may have had similar experiences. It will also highlight areas for improvement to better support women and children during future pandemics or other public health emergencies. One woman shared her experience of giving birth during the lockdown period and the subsequent death of her baby. She recounts how a medical student prescribed a drug for high blood pressure during pregnancy that she perceived was unsuitable for pregnant women. She perceived that despite knowing about the potential risks to her baby, the doctor still prescribed the drug.

*My experience during that time [lockdown] was bad. I had my baby during that period [lockdown]. I was prescribed a drug because my BP was high by a medical student doctor*,* not a consultant. As a pregnant woman*,* you will not take that drug when you are pregnant. Even when you deliver*,* you will not take it because the baby might take it through breast milk. When I was attending antenatal care (ANC)*,* nothing was wrong with him*,* the baby was gallant. I couldn’t even imagine anything would happen to the baby*,* but he died. The doctor said that the liver was too immature to carry the load*,* so the baby died. Even the birth weight was also low. [Chinwendu*,* Businesswoman]*

For Chinwendu, attending antenatal care visits assured her of the health and well-being of her baby. In describing her ANC experiences before delivery, Chinwendu reaffirms her trust in the health system and her belief that attending ANC would ensure not just a safe delivery but the best health outcome for her child. She uses the word “gallant” to describe her perceived state of her child in-utero, which is an adjective used in Nigeria to connote strong and healthy. However, despite attending all the visits and being reassured that everything was fine, her baby died shortly after birth. She uses “student” and “consultant” to describe the level of expertise of the health professional who prescribed the drug to her. It suggests she perceived that the medical negligence might have been avoided if a more experienced doctor attended to her. Chinwendu shared her story as an example of a negative experience of healthcare seeking for herself and her child during the pandemic. In her account, the fact that someone with less experience than a consultant doctor attended to her was due to COVID-19. In her description, to support her assertion, she recalled knowledge of the drug from her experience in her previous pregnancy post-pandemic.

This experience highlights the fragility of trust in healthcare systems and the potential for devastating consequences when that trust is broken. She recounts her experiences with the emotional weight of the loss. Her description that nothing was wrong with the child indicates a struggle to come to terms with the tragedy. She noted that her current pregnancy is better managed as she is attending ANC in a different hospital, and they manage her BP better. She explains that her experience did not prevent her from returning to the hospital. Ultimately, Chinwendu attributed her loss to an act of God. Her willingness to “accept” the death of her baby as fated indicates her need to trust the health system which is restored by changing hospitals where she perceives she is receiving better treatment. Chiwendu’s reasons that the medication prescribed by the student doctor may have been a side effect of the pandemic since she perceives her care is now better managed post-pandemic, and that the pandemic times were a challenge for healthcare workers who were especially strained with workload and shortages. Therefore, likening it to a natural event where no one can be blamed.

*Let me just accept that it’s how God wants it. If not*,* I know that simply living would be a problem. I am pregnant again*,* and by God’s grace*,* I will deliver safely. I am not taking the BP drug anymore*,* but my new hospital is checking my blood pressure every time I go for ANC. I am also trying to be a more active woman*. [*Chinwendu*,* Businesswoman]*

To further highlight and understand the impact of the pandemic on women who were pregnant and delivered during the pandemic, another woman, Chichi shared her hospitalization and pregnancy experience during the pandemic. She was pregnant during the lockdown, and two months into her pregnancy, she became ill and had to go to the hospital for testing. The test showed she had malaria and typhoid, which was treated, but she did not feel better. She described a persistent headache that continued even after receiving the usual treatments given to pregnant women. She eventually went to a referral hospital, where they discovered she had COVID-19.

*I was pregnant with my last baby during the lockdown. The first two months of the pregnancy went fine*,* but during the 4th and 5th months*,* I became ill. I thought it was an iron deficiency*,* but the test revealed that I had malaria and typhoid*,* which I was treated for. However*,* even after the treatment*,* I continued to have headaches at home*,* and there was nothing they could do as the usual drugs for pregnant women weren’t working. Eventually*,* I had to leave the private hospital to the teaching hospital for further testing*,* and that was when they discovered that I had COVID-19. At that point*,* I was in my 3rd or 2nd trimester*,* and I was hospitalized. [Chichi*,* Businesswoman]*

She described in detail her hospitalization experience during pregnancy, where she faced respiratory distress that necessitated oxygen therapy, and the hospital recommended her placement in quarantine to continue her treatment. She recalled her difficulty breathing, requiring oxygen, and the hospital’s intention to place her in quarantine while continuing oxygen therapy. However, her husband refused to allow her to be quarantined. She went home and was cared for by family and friends for two weeks after her hospitalization. She described returning to the hospital to continue ANC and being informed that two other pregnant women who came afterward died in quarantine.

*During my hospitalization*,* I had difficulty breathing*,* and I could only breathe normally when I was on oxygen. I stayed in the hospital for three to four days*,* and they recommended that I be quarantined for two weeks. However*,* my husband refused*,* and we went back home. I continued taking routine drugs*,* vitamin C*,* and resting for an extra two weeks*,* and only my sister-in-law and a close friend visited me at home. My husband tried to get oxygen so I could continue using it at home but could not. After two weeks*,* I noticed an improvement in my condition*,* so I went back to the hospital to continue my antenatal care. When they saw me*,* it was like a miracle because two pregnant women who had come after me had both died where they were quarantined. I delivered safely in the 9th month. [Chichi*,* Businesswoman]*

In her perception, she feels that her husband’s refusal to quarantine her potentially protected her and the baby’s health, which was at risk. When she described her husband’s effort to procure oxygen to use at home, she is acknowledging he was actively trying to find ways to care for her. She uses the word “only” to further stress that the family limited contact with outsiders in the two weeks she was ill. She notes that her condition improved after the two weeks at home, and she returned to continue antenatal care. Her use of the word “miracle” to describe her survivorship and comparison of her survival to the death of other women in her condition during that period draws attention to the exceptionally high risks faced by the many women needing routine healthcare during the pandemic.

In Martha’s case, she described significant differences in hospital treatment between her first pregnancy in 2018 and her second pregnancy in 2021. She described registering for ANC 16 weeks into her pregnancy, while during the pandemic, she went to register at 25 weeks and was told by health professionals that she could not register without any visible symptoms of complication. She felt that health workers were trying to minimize the number of people in the hospital with whom they came in contact.

*There’s much difference between the time when I had my first baby in 2018 with this one in 2021. There are differences in hospital treatment. For my first child*,* I registered for antenatal visits at 16 weeks*,* but for this one*,* I was already 25 weeks gone*,* and I was told*,* ‘If you are not sick*,* there is no complication*,* or you are not bleeding now if you do not have all of these*,* please go and come back next month.’ I believe they were trying to reduce the number of people coming into contact with the hospital. [Martha*,* No paid employment]*

She questions the rationale behind such a decision to ask pregnant women to return only if they have complications “*But*,* why would the pregnant woman come to be registered for ANC and tell her to come back?”* Her question seems to indicate that she is dissatisfied with the new system of registering for antenatal visits and being asked to return the next month only if she has complications. She described her labour and how she prayed to deliver in the night to avoid encountering people in the hospital.

*When I was close to having my baby*,* I remember always praying to go at night because I didn’t want to go to the hospital when it was too busy with many people. On the day my son was born*,* there were lots of student doctors in the hospital*,* and in the labour room*,* there were not just women in labour*,* but also doctors and nurses. [Martha*,* No paid employment]*

She described the presence of a mix of health professionals attending to her in the delivery room. She noted the presence of medical students, using the word “lots” to describe the number of medical students attending to her. She defended her apprehension of medical students with, “*I didn’t want to have a bad experience not because I didn’t want them around but because of what was happening in the world at that time.”* She appears to feel it may result in a “bad” delivery experience. She described her fear and wanting to have the baby at night to reduce contact with people and still get the doctors to observe for complications before discharging her in the morning.

*I wanted to have my baby at night so that I would be observed for six hours and then be discharged. When I had my first baby*,* they observed me for eight hours before discharging me. However*,* during and after the COVID outbreak*,* the observation period was cut down to four hours. After four hours of observation and no complications*,* they would ask you to leave. So*,* when I had my second child at night*,* I was discharged very early in the morning*,* around 8 AM*,* after being observed for four hours and with no complications. [Martha*,* No paid employment]*

Martha perceived that having her baby at night would give the health workers more time to observe her. She compared the observation time between her delivery in 2018 and 2021. She described a reduced time from 8 h to 4 h of observation because of the pandemic.

For Chinwendu, Chichi, and Martha, the maternal care they received during the period did not influence their decision not to seek care; instead, it reinforced the need for medical care during pregnancy. From their perception, there seems to be a general apprehension toward medical students and the quality of care they provide.

The stories of Chinwendu, Chichi, and Martha not only validate the experiences of many women during the pandemic but also serve as a crucial call to action for enhancing healthcare practices and policies to ensure better support for women and children during any future public health emergencies. Their experiences are a testament to the resilience of women and the urgent need for a healthcare system that upholds their health and well-being, even in the face of global health crises. Ahead of the next global public health emergency, the government needs to improve its preparedness and response strategy on how pregnant women are treated during this period. The experiences these women highlighted show a clear need for specific guidelines tailored to managing pregnant women during such emergencies. Developing adaptable, sensitive policies that can respond to the evolving nature of public health threats is, therefore, essential. These policies should ensure that every woman, especially the most vulnerable, continues to receive care that is both appropriate and sensitive to their specific needs.

## Discussion

This study explored women’s encounters with healthcare and their lived experiences during the pandemic in Ebonyi, Ogun, and Sokoto states of Nigeria. Our study highlights the importance of understanding how individuals experience and make sense of their world to gain insight into the impact of a global event like a pandemic on health behaviour using an interpretive phenomenological approach. The findings revealed three themes: (1) the transformations of personal health behaviour in response to the pandemic; (2) the pandemic as a temporal equalizer for marginalized individuals; (3) the impact of the COVID-19 pandemic maternal health care during the COVID-19 pandemic.

Our study contributes to existing research on the impact of COVID-19 on maternal health-seeking behaviour in Nigeria [52–54]. Over the past two years, many studies have focused on understanding and unraveling the often-unintended consequences of the pandemic on public health systems [55–59]. While these studies provide invaluable insights, our research enriches the existing body of research by applying a feminist lens to explore these effects more intensely. This approach highlighted the specific challenges women experience during the COVID-19 pandemic, emphasizing women’s adaptability and resiliency in times of health emergencies. Focusing on women’s lived experiences, we advocate for a more person-centered approach to pandemic preparedness and response. More specifically, in understanding the gendered effect of the COVID-19 pandemic, our study provides decision-makers and program managers information to develop and enhance existing policies against future pandemics. Additionally, our study highlights the importance of incorporating gender perspectives in future pandemic planning and response, which has been under-considered in previous models of health emergency management.

Common to the accounts of some women in this article are women’s experiences of the pandemic as changing their experiences of healthcare seeking as distinct from their norm during the pandemic’s peak. These differences, however, did not seem to factor into their decision to seek or delay care for themselves or their family. While these experiences suggest that the pandemic disrupted maternal healthcare and highlights the need for interventions to improve access to care for pregnant women in future plans, there appears to be an unwavering trust in healthcare systems for some of the women. These findings contradict similar findings and projections made at the beginning of the pandemic that maternal and child health services will be disrupted, with a potential impact lasting several years [7, 60–64]. This study provides an alternative perspective from which to view the effect of the pandemic on maternal and child health and the positive lessons that it potentially provides for future pandemic preparedness.

Our study shows that the COVID-19 pandemic has led to a significant shift in health behaviours among women. The finding reveals how the pandemic has influenced women’s daily experiences and practices, leading to new ways of being in the world. Several women described having an increased awareness of personal hygiene practices and the importance of regularly washing hands and using nose masks to prevent the spread of infections. From their responses, these women internalized the importance of personal hygiene practices, which is reflected in the language they use to describe their behaviour change. They use words such as “mindful,” “careful,” “regularly,” and “still” to indicate an extension of behaviours aimed at reducing risks of infection learned during the pandemic beyond its peak. The adoption of these behaviours has become a critical component of daily life for many of these women, resulting in a potentially positive impact on their overall health. The women note that they have learned about hygiene practices at work and from others around them, suggesting that the pandemic has created a collective effort towards maintaining personal hygiene. This shared experience has likely contributed to the adoption of these behaviours among women. Overall, this finding suggests that women perceive these behaviours as new and a direct consequence of the pandemic. It suggests that on a broader level, the pandemic has also brought about a collective sense of caution and awareness that has impacted how people interact with their environment and each other.

We found that the pandemic has brought to light the specific needs and vulnerabilities of individuals with disabilities in emergencies, including access to healthcare. Two women in our study self-identified as having disabilities, and their experiences highlight how societal attitudes toward disability can intersect with pandemic realities. Despite contributing to society through their businesses and education, these women’s accounts underline their feelings of being devalued, unappreciated, and feared by society. For these women living with disability, the pandemic provided some relief from social stigma. Women perceived the treatment from health workers and others as improving because of the pandemic. They expressed that getting to the hospital was easy since they did not have to contend with the stares of onlookers on the street and in the hospitals. It is essential to recognize that the pandemic has disproportionately affected people with disabilities, who may have been excluded from the mainstream conversations around pandemic preparedness and response. Stigma and discrimination can create barriers to healthcare access, which can be compounded in emergencies like a pandemic. It is crucial to consider disability-inclusive approaches to health planning and interventions to ensure that individuals with disabilities are not left behind. However, the findings from this study indicate that often environmental disruptions such as pandemics may not increase vulnerabilities for some individuals but paradoxically may provide relief from their daily lived experiences.

Our findings confirm that the pandemic had a significant impact on maternal health care, as seen in the experiences of Chinwendu, Chichi, and Martha. While all three women experienced childbirth during the pandemic, their specific experiences with hospital treatment and care differed significantly. Chinwendu’s experience highlights the fragility of trust in healthcare systems during environmental disruptions and the potential for devastating consequences if that trust is broken. Despite attending all the antenatal care visits and being reassured that everything was fine, her baby died shortly after birth. Chichi’s experience shows how the pandemic has affected access to care and the challenges faced by pregnant women who contract the virus. She had difficulty breathing and required oxygen therapy, which her husband tried to provide at home but was unavailable. The hospital recommended that she be quarantined to continue her treatment. However, her husband refused, and she had to rely on family and friends for care after her hospitalization. Fortunately, she recovered at home. Her story, however, and her attribution of her survival to a miracle given other women who abided by healthcare professionals’ recommendations for quarantine in hospital died, underlines the increased risks women may face during pandemics. In contrast, Martha’s experience differs from Chinwendu and Chichi in several ways. Firstly, Martha’s experience of hospital treatment during the pandemic is characterized by delays in accessing antenatal care, as she was told to come back next month if she did not have any visible symptoms of complications, whereas Chinwendu and Chichi were able to access ANC despite the pandemic. Secondly, Martha was apprehensive about encountering many people in the hospital during her delivery and prayed to deliver at night to reduce contact with people, while Chinwendu and Chichi did not express similar concerns. Thirdly, Martha perceived a reduction in the observation time after delivery from 8 h to 4 h due to the pandemic, which attributes to not contracting the virus after childbirth while Chinwendu did not mention any changes in the observation time, and Chichi did not comment on this aspect of her experience. The reduction in the duration of hospitalization after childbirth is a policy that can potentially have both positive and negative impacts. One advantage of this policy is the reduced exposure to contagious diseases for both the mother and the newborn. However, the shortened duration of hospital stay during emergency situations may increase the likelihood of complications that may have been detected with adequate monitoring and observation during the postpartum period.

This study has two fundamental limitations. One, the sample size is too small for generalization. While the women interviewed in this study were drawn from three geopolitical zones, Nigeria’s diversity is such that a generalizable study requires a sample from over 450 ethnic groups. However, we must note that while it is too small to be representative, it is still useful to elicit experiences that may inform some theories and generalizations. Two, Nigeria is a diverse country with over 450 languages spoken. Communication can be challenging when conducting research in areas where English is not widely spoken or understood because there is a tendency to lose the essence of meaning in translation. This study was time constrained and lacked the resources to conduct such a large-scale study. The key strength of this study is its exploratory approach and the utilization of IPA theory and philosophy to gather extensive and profound insights into the lived experiences of mothers. This research approach is critical in creating new knowledge that can form a foundation for formulating a novel intervention model.

As we move forward from the pandemic, it is important to recognize the intersection of disability and pandemic preparedness and ensure that disability-inclusive approaches are implemented in emergency situations. While the health system outside pandemics have not adequately addressed the health access challenges of people living with disabilities, it is important to recognize that during pandemics and outbreaks people living with disabilities may experience some sense of relief given the reduced contact with people which paradoxically provides some benefit to those who suffer from stigma related to social contact. It is evident from the experiences of the participants that there is still a lot to do to promote positive attitudes toward individuals with disabilities. By doing so, we can work towards a more equitable healthcare system that meets the needs of all individuals, including those with disabilities.

Further research is needed into the impact of emergencies on people with disability’s access to health. The changes in the provision of maternal healthcare services, such as the reduced duration of hospital stays after childbirth, which potentially affected the quality of care received by the women, will require an innovative approach, such as leveraging technology to address access issues during the pandemic. For example, health facilities could implement virtual antenatal care visits to reduce the risk of exposure to infections while ensuring that pregnant women receive necessary care. Further investigation is required to ascertain the impact of this policy on the survival of postpartum women and babies. While awaiting the outcome of such studies, it is pertinent for extant policies to incorporate distinct directives that stipulate the specific circumstances that warrant brief hospital stays during pandemics. Such interventions can improve maternal health outcomes and reduce the risk of adverse pregnancy outcomes, even during a pandemic. It is, therefore, necessary for stakeholders to explore specific plans at different levels of the health system to address the unique challenges at each level.

## Conclusion

We can conclude that the experiences of pregnant women in Nigeria during the COVID-19 pandemic were affected in diverse ways. These stemmed from the pandemic’s impact on recommended healthcare practices, the healthcare system, and the daily landscapes shaped by norms of disadvantage and discrimination that women, and particularly women socially marked as having disabilities, must navigate to seek healthcare. COVID-19 related challenges faced by the women in this study include delays in seeking antenatal care, reduced access to healthcare facilities, and changes in the quality of care received during childbirth. If the current pandemic has revealed anything, stakeholders need to develop future strategies to mitigate the effects of pandemics on maternal healthcare services, especially for women in low-income communities who may not have access to private healthcare facilities.

### Electronic supplementary material

Below is the link to the electronic supplementary material.

Supplementary Material 1

Supplementary Material 2

Supplementary Material 3

Supplementary Material 4

Supplementary Material 5

Supplementary Material 6

Supplementary Material 7

Supplementary Material 8

## Data Availability

The tools used in this study are available; authors interested in using them can contact the corresponding author.
