# Supplementary material for: “It’s what we perceive as different”: an interpretative phenomenological analysis of Nigerian women’s characterization of their health during the COVID-19 pandemic
Source: BMC Womens Health. 2024 Jul 18;24:409. doi: 10.1186/s12905-024-03259-w (PMC11256442; doi:10.1186/s12905-024-03259-w)
Supplement: Supplementary file 1 — Supplementary Material 1 [file 12905_2024_3259_MOESM1_ESM.pdf]

Igbekale iforowanilenuwo.

Isawari iriri awon obinrin to nwa itoju nigba ajaakele arun tokari-aye ni orile-ede Nigeria:

Ibẹẹrẹ:

E seun. Ki a to bẹẹrẹ, ma fẹ ki ẹ gbami laaye lati gba ohun yin silẹ. Ighohun silẹ yii yo fun mi ni anfani lati kọ gbogbo ohun ti e ba sọ silẹ bi ẹ se sọ. Mofe ki o da yin loju pe, emi ati alabojuto mi ati ile ẹkọ giga Univesity of Western nikan ni yoo ni le lo igbohunsile yii, a o si parẹ leyin odun 7. O ni anfani lati ma fi ohun ẹ silẹ nigba ti o ba nfẹ nigba ifọrọwanilẹnuwo. Pẹlu o le kọ lati dahun awon ibeere ti o ba fe. Ni akoja o le yan lati ma tẹsiwaju pẹlu ifọrọwanilẹniwo ki wọn si pa gbogbo ohun ti a ba ti gba kale rẹ. Ri daju pe ogba iwe alaye ati iwe ijerisi pe ki afi oro wa o lenu wo ki a si gba ohun ẹ silẹ.

Ibeere:

1) Sọ fun mi nipa ara rẹ.
   Iwadi:
   a) Abẹlẹ ọrọ
   b) Idile
   c) Abele ẹkọ

2) Se o le sọ awọn iriri rẹ nigbati ara re koda to o si fe gba itọju?

   a) Tani iwọ yo ba sọrọ ti o ba nfẹ iranlọwọ?
   b) Ibo ni wa lo ti o ba nfẹ iranlọwọ (ibasepọ, inawo, imọlara).
   c) se o ni ero wipe wọn fun ẹ ni iranlọlwọ? Ni ọna wo?
   d) Nje ẹnikẹni wa ti o fẹran lati tọ lọ fu imọran (oko rẹ, arabinrin rẹ ,iya rẹ, aladuugbo rẹ)? Kini idi ti o fi fẹ lati gba imọran lọwọ wọn.
   e) Se bi o se nwa itọju yato ni gba ajakaalẹ arun tokari-aye.

3) Ṣe o le sọ boya bi o se nwa ọrọ ilera fun iwọ tabi ọmọ rẹ nigbati ẹ ba nṣe aisan sṣe jọ ati bi oṣe yatọ nigba ajakale arun tokari- aye?
   a) Tani iwọ yo kan si ti o ba nfẹ iranlọwọ?
   b) Ibo ni iwo yo lo ti o ba nfe iranlọwọ (fun ibaragbe, owo, imọlara,)
   c) Se wọn wulo?
   d) tani ti iwọ nifẹ lati kani si gan (ọkọ, arabinrin rẹ, iya, aladuugbo)?
   e) Se bi o se ngba itọju fun ọmọ re yatọ nigba ajakale arun tokari-aye? (bawo)?

4) Nje o le sọ bi ati gba itọju ṣe ri fun alaboyun ni ile iwosan nigba ajakale arun COVID 19

a) Ṣe o bi ọmọ nigbati ajakale arun COVID 19 wa lode? Ṣe oyun akọkọ rẹẹ ni yi? Bi ko bari bẹẹ se iyatọ wa si igbati kosi arun ajakaye COVID 19 ti o bimo? Nje inira kan kan wa? So fun mi nipa re
b) Bi ki ba se oyun alakọkọ re, ona wo iriri ọmọ bibi rẹ se yatọ si igbati kosi COVID 19?
c) Ṣe o gba itoju lowo awon osise ilera? Bawo ni ibasepọ yin ti ri?
d) Bawo ni eto itọju won se ri?
e) Ki lo wulo, kini ko wulo (rii wipe o beere alaaye lori bawo/ idi ti ofi wulo ati ti ko fi wulo) se o lero wipe awọn osise ilera yii gbo ni agboye?
f) kini idi ti o fi lọsi ile iwosan?
g) ayipada wo lo fẹ?

5) Ṣe o le sọ funmi bi igba Kankan ba wa ti ara re ko da ti o si pinu lati ma lo si ile iwosan gẹgẹbi eniti o ti balaga?
a) kini idi ti o pinu lati ma lọ?
b) kini nka ti yo din inira a ti lọ si ile iwosan ku?
c) ayipada wo lo fẹ?

6) Nję o le ṣe alaaye iriri ailera to je ipenija nla fun ọ nigbati arun ajakaye COVID 19 wa lode?
Iwadi:
a) Nję o le se alaaye bi ṣe bori rẹ?
b) Ṣe o le ṣe alaaye bi o se dajukọ ipenija wonyi?

7) Se o le so igbagbọ re ati iye re nipa eto ilera?
Iwadi:
a) kini itumo ki eniyan wa ni Alafia?
b) bawo ni o se nṣe eto bi iwọ ati awon ọmọ re wa ni Alafia?
c) bawo ni igbagbọ ati iye re nipa eto ilera se yato lati igbati COVID 19 ti de?

8) Nje o le sọ ipinnu ti iwọ ti se nipa ọrọ ilera re lati bi osu 6 sẹyin?
a) Se o le se alaaye bi iriri se dara si lati igbati wọn ti yo ihamọ kuro?
b) Se o le sọ fun mi boya ibasepọ rẹ pẹlu awọn osise ilera dara si lati osu mefa si asiko yi?

With seal of Dr. Ndubuisi Ahamefula Certified Professional member number 147 Nigerian Institute of Translators and Interpreters (NITI)
Lecturer, Department of Linguistics, Igbo & Other Nigerian Languages, University of Nigeria, Nsukka.
