# Supplementary material for: “It’s what we perceive as different”: an interpretative phenomenological analysis of Nigerian women’s characterization of their health during the COVID-19 pandemic
Source: BMC Womens Health. 2024 Jul 18;24:409. doi: 10.1186/s12905-024-03259-w (PMC11256442; doi:10.1186/s12905-024-03259-w)
Supplement: Supplementary file 2 — Supplementary Material 2 [file 12905_2024_3259_MOESM2_ESM.pdf]

Itọni sọna ẹgbẹ idajukọ fun ijiroro.

Ohun elo:

- Igbohun sile

Osise:

1. Oluṣeto (Onkawe Ph.D)
2. Oluranlọwọ iwadi (Jhpiego)

Ẹgbẹ idajukọ yo jẹẹ awọn obinrin ti o ti bi mọ ti ko ju odun marun lọ ti ọjọ ri wọn sii wa larin 15 si 49. Ẹgbe idajukọ kọkan ko gbọdọ ju eniyan mejọ lọ. Awọn olusetoo se ifihan arawọn wọn o ṣe alaye idi ti wọn fi ko wọn jọ. Won yo ṣe alaye ni ṣoki ohun ti ẹgbẹ yo ma se. Alaye naa yo si tele ẹda to wa ni isalẹ:

Ẹkaabọ! Adupe pe e fi owo si iwe asẹ ati bi ẹ ṣe pada wa si ibi ni eni. A nse ipade yii lati jiroro lori iriri yin nigbati ẹ ba nwa itọju fun ailera yin lati igbati ajakalẹ arun tokari-aye ti de. Gbogbo ijiroro wa ni yow a ni ipamo. Ma beere pelu ibeere pe ki e se alaye ni soki iyikeyi iriri ti eni bi e se nwa itoju fun ilera yin. Kini iriri yin nigbati e ko wa itoju, kini idi ti eko fi wa itoju. A o tun wa ṣe ijiroro lori iriri yin yekeyeke. Ti o ba ni iyikeyi ibeere ti ẹ ko fe dahunsi, kosi wahala. Ẹ jowo ẹma fi nkankan pamo lara iriri yin.

Gbogbo akoko ti a ma lo ko le ju wakati meji lọ. A pese nkan ipanu jijẹ ati mimu fun yin ni akoko yi. Bi ẹ ṣe gbo ni ana nipa iwe igbanilaaye a o gba ohun yin silẹ, sugbon ako ni so yin pọ mọ ohun ti a gbakalẹ. A fe ṣe igbohun-sile ijiroro yii ki a leni akosile to se deede. O le pinu lati kuro ninu ẹgbẹ idojuko in igbakigba to ba fẹ. Sibẹsibẹ mọ wipe koni rọrun lati yọ awọn oro ti o ti sọ ki o to di wipe o kuro. Ranti wipe ki i ṣe didan ni ki o dahun awọn ibeere ti ko fi ọ lọkan balẹ tabi ti o fẹ dahun si. Nitoripe awọn iyoku ti ẹ jo wa ninu ẹgbẹ ijiroro mọ ọ , oluseto ko ni le jẹri pe won yo bọọwọ fun asiri ẹgbẹ lati ma sọ sita. Iṣẹ ti a nṣe yi jẹ apakan iwadi ti a nṣe ni ipinle 3: ipinlẹ Ebonyin, ipinlẹ Ogun ati ipinlẹ Sokoto. Asi ni ireti pe awẹn iriri ati irisi yin yo wulo fun ṣiṣe eto itọju ailera fun awọn obinrin ati awọn ọmọ wọn ni ijọ iwaju. Ki a to bẹẹrẹ a fẹ ki ẹ buwolu iwe iganilaaye pe gbogbo alaye ti a ṣe ni oye yin daradara ati wipe ẹ gba lati fi gbogbo nkan ti a ba sọ ninu egbe yi pamọ.

1) Oluseto a bẹẹrẹ ijiroro pelu gbolohun yi: bayi , ni ṣoki, ki ẹnikan ṣe alaaye bi o se lọ kan si awọn olosise ilera? Tani yo bẹẹrẹ? Gba gbogbo awọn to ba fẹ sọrọ laaye. Akoko: 20 iseju.

2) Lẹyin ti gbogbo wọn ba ti sọ itan wọn tan, tọkasi pe o fẹ gbọ awọn itan bi melo kan daradara si. Oniwontunwonsi a si yan awọn itan bi marun ti e dojuko. Awọn itan gbọdọ ni awọn nkan wọnyi:
Itan na gbọdọ ni orisirisi alaaye nipa orisi eto ilera ti wọn lọ fun, ati iriri to dara tabi ti ko dara. Eleyi ni kan ni ijiroro ti yo gun ju akoko rẹẹ yo si je wakati kan gbako.

Gbogbo awọn itan ti ẹ sọlo dun daradara. Mo fẹ se akiyesi si di ẹ ninu wọn. Ma bẹẹrẹ pẹlu itan X, X jọwọ ṣe o le ṣelaye iriri rẹ lẹkurẹrẹ. Bi X ṣe nsọ itan rẹ mo fẹ ki gbogbo yin fi ara yin si ipo X kini yo jẹ ero yin ti o ba ṣe ẹyin ni nkan na sẹlẹ si. Leyin ti X ba ti tun itan rẹ sọ tan, ma beere awọn ibeere kan fun itọnisọna. Ma wa sọ fun gbogbo awọn ti o nkopa ki wọn beere ibeere ki won si sọ ero wọn lori igbesẹ X si awon iriri rẹ. Lẹyin ta bari ijiroro lori iriri X, a o beere itan miran. Ma fẹ ki a kaju itan mẹrin si marun ni ona yi.

3) leyin ti eni akoko ba ti so itan de opin, oniwontunwonsi a tun beere awon ibeere lowo eniti o nso itan ki o le soro siwaju si:
a) jowo so fun wa nipa ibi ti o ba olosise ilera? Fun apeere se ile iwosan ijoba ni tabi aladani?
b) Se ibe ni ile iwosan ree?
c) Igba wo ni isele naa sele?
d) Bawo ni awon olosise ilera ibe se se itoju ree?
e) Bawo ni ose ri ibe sii.
f) Iru iyipada wo ni iwo yo fe to ba fe ki iriri re yato, yala ara re da tabi koda?
g) bayii eyin iyoku e so ero okan yin si itan X.

4) Ibi ni ipari apa ijiroro.
Eyi ni ibi ti a ti ma fi opin si ijiroro. Ọpo ni o si to sọ awọn nkan ti o sẹjẹ bi ẹ ṣe nwa itoju, sugbon a ro boya ari ẹnikẹni ti ko lo fuh itoju nigbati ara re koda. Ṣe ẹnikẹni lara yin to ni iru iriri yi le so fun wa nipa rẹ

5) Ri daju pe alaaye won dahun ibeere yii:
Kini idi to fi lo fun itoju?

6) Awọn Oluseto a ri pe won kiyesi awon ti ko kopa. A le fi kadi ti a ya so to ati iwe to ni aworan bi owo fun aini owo lowo, okunrin fun oko, obinrin arugbo fun iya oko, iwaju ibuso fun okowo fun awon ti koba toripe oje inira fun won lati soro. So fun idi kokan ki won se alaaye pelu awo aworan yii idi ti won fi lo fun itoju.

7) Lati mu asiko wa si opin awon oluseto a dupe lowo fun ikopa won ninu egbe idojuko. Awon itan iriri wonyi dun gan ni won si kun fun oro iyanju. Adupe lowo fun ikopa yin.

With seal of Dr. Ndubuisi Ahamefula
Certified Professional member number 147 Nigerian Institute of Translators and Interpreters (NITI)
Lecturer, Department of Linguistics, Igbo & Other Nigerian Languages, University of Nigeria, Nsukka.
