# Supplementary material for: “It’s what we perceive as different”: an interpretative phenomenological analysis of Nigerian women’s characterization of their health during the COVID-19 pandemic
Source: BMC Womens Health. 2024 Jul 18;24:409. doi: 10.1186/s12905-024-03259-w (PMC11256442; doi:10.1186/s12905-024-03259-w)
Supplement: Supplementary file 3 — Supplementary Material 3 [file 12905_2024_3259_MOESM3_ESM.docx]

**Usoro Onye Ntụziaka nke Otu Nlegara Anya**

**Ihe ndị Achọrọ:**

- Ụda Rekọdụ

**Ndị Ọrụ:**

1. Onye Ntụzi aka nke mbụ ( Onye na-achọ ịturu ugo Ph.D.)
2. Ndị enyemaka nchọcha (Jhpeigo)

Ndị otu nlegara anya ga-abụ naanị ndị nwaanyị nwere ụmụaka na-erubeghi afọ ise (5) ma ha onwe ha ga-anọ agbata afọ iri na ise (15) ruo afọ iri anọ na itoolu (49). N’ime otu nlegara anya ọbụla, agaghị enwe ihe karịrị mmadụ asatọ. ndị ntụziaka ga-akọwa onwe ha ma kọwaa ihe mere eji kpọkọta otu nlegara anya. Ha kwesịrị inye obere nkọwa maka ka aga-esi hazie otu nlegara anya Nkọwa a ga-adị etua:

**Unu abịala! Ekele m gị maka ibinye aka n’akwụkwọ fọmụ nkwenye isonye ma weghachite ya n’nzukọ a. Anyị zukọrọ ịkparịta ụka gbasara ihe ndị une gabigara mgbe unu na-achọ nlekọta ahuike kemgbe gbasa ọrịa koro. Ihe anyị ga-akpa maka ya ebea ga-anọgide dịka ihe nzuzo. M ga-amalite site ị si unu kọwaa na nkenke ihe ọbụla unu gabigara mgbe unu na-achọ nlekọta ahuike. M ga-asịkwa unu kọwaa mgbe unu nwere nkpebi na agaghị achọ nlekọta ahuike nakwa ihe mere unu ji new ụdị mkpebi a na ya achọghị nlekọta ahuike. Anyị aga n’ihu kpaa banyere ụfọdụ ihe ndị a une gabigara n’uju. Ọbụrụ na enwere ajụjụ ị chọghị ịza, nke a dị mma, nsogbu adịghị. Biko, kọọrọ anyị maka ihe ndị ị gabigara nye ọha mmadụ. . marakwa na enweghị ọzịza nke dị mma maọbụ nke dị njọ, ha niile dabara adaba.**

**Nkebi nke a niile nwereike iwere ihe dịka awa abụọ. Anyị ga-enye unu ihe ọṅụṅụna ihe ntatụ achịcha bekee mgbe nke na-aga n’ihu. n’oge a. Dịka unu nụrụ ụnyahụ mgbe anyị kpara banyere akwụkwọ nkwenye isonye Anyị ga erekọdụ nkebi niile, mana onweghị ihe I kwuru na teepụ nke a ga-eme ka amata na ọ bụ gị kwuru ya. Anyị na-erekọdụ nkebi niile nihi na anyịchọrọ inwetacha mkparịta ụka niile kaọdị na-ewepụgị ihe ọbụla. Ọ dị gị n’aka ikpebi na ị chọghịkwa isonye n’otu nlegara anya oge ọbụla ị chọrọ. Kaosiladị, mar na ọgaghị adịcha mfe ihichapụ okwu niile I kwur tupu I kpebie ịpụ. Cheta na I nwereike ị hapụ ịza ajụjụ ọbụla nke adịghị gị mma n’obi maọbụ nke ị chọghị ịza. Akara njirimara gị bụ bụ nke ndị ọzọ so n’otu nlegara anya macha, mdị nchọcha anaghị enye gị nchekwube na ndị ọzọ nọ n’otu nlegara anya ka ekpuchi ihe ndị emere ebea dịka ihe nzuzo. Ọrụ antị na-arụ ebea taa bụ otu n’ime nchọcha anyị na-eme na steeti atọ: Steeti Ebonyi, Ogun, na Sokoto. Anyịna-enwe olile anya na ihe ndị ị gabigara nakwa etu isi kọọ ya ga-enye aka ime iwu na atụmatụ ahuike maka nd nwaanyị na ụmaụakaị. Tupu anyị amalite, anyị ga-achọ ka ibinye aka n’akwụkwọ ọụ nkwenye isonye ọzọ I ji kwesie ike na ị mara nke ọma ihe niile anyị kọwara na ị kwere ị dowe ihe niile ekwuru n’otu nlegara anya a dịka ihe nzuzo.**

1. Onye ntụziaka ga-amalite mkparịtaụka site n’ikwu ahịrịokwu mmalite ndị a.The facilitator then:

**Ugbua, na nkenke, biko onwere onye nwereike kọwara anyị ihe mere mgbe ọ gara na nke oonye nlekọta ahuike? Kedụ onye ga-ebu ụzọ bido?**

(Hapụ ọtụtụ ndị mmadụ enwereike ị nagịde n’oe enwere ndị nwere mmasị ikwu okwu ka ha mee nkea. Oge a na-ele anya bụ ihe dịka: Nkeji iri abụọ (20))

1. Mgbe onye ọbụla kọchara akụkọ ya, mee ka amara na ị ga-achọ ileba anya n’ụfọdụ akụkọ n’uju ya. Onye nchịkọcha da-ahọta ihe dịka mmadụ ise ileba. A ga-agbasoro usoro ndị a maka ịhọpụta akụkọ ndị ahụ: akụkọ ndị ahụ ga-enwerịrị ihe dịiche iche banyere nlekọta ahuike dị iche iche nakwa ka ihe ha gabigara si dị mma maọbụ dị njọ. Nkebi otu nlegara anya a bụ nke kacha dị ogologo bụrụkwa nke ga-ewe ihe dịka otu awa.

**Akụkọ unu kọrọ dịcha mma. Achọrọ m ka anyị lebe anya n’ụfọdụ akụkọ n’uju ya.. Achọrọ m ka anyị malite n’akụkọ X. X, biko, I nwerike ịkọwara ọzọ ihe ịgabigara n’uju ebe ọ dị nkenke. Aga m achọ ka onye ọbụla nọ n’otu a tulee uche n’ihe mere X na ka ha si nweee mmetụta gbasara ọnọdụ X. Mgbe X has kọrịchara akụkọ ya , m ga ajụ ajụjụ ụfọdụ maka nghọta ya nke ọma. Emechaa, ọ ga-amasị m imepe mkparịta nye ndị otu a niile ka ha jụọ ajụjụ ma tinye ọnụ n’okwu n’ụzọ ọbụla gbasara ihe ha chere maka ihe gabigara. Ka anyịna-emecha mkparịta ụka gbasara ihe X gabigara, anyị ga-aga n’ihu kpaa maka akụkọ ọzọ. Ọga amasị m ka anyị lebe anya na ihe dịka akụkọ anọ maọbụ ise akọrọ ebe a taa n’ụzọ yiri nke mbụ. X, biko ị nwereike ịmalite?**

1. Mgbe akọchara akụkọ nke mbụ, onye nchịkọta ga-eme ka ndị na-aza ajụjụ nyekwuo ihe ndị ọzọ n’iji ndị a, maọbụrụna na dị mkpa maọbụrụna onye ọza ajụjụ ekwughị maka ha:
2. **Biko kọkwuoro m maka ebe ị hụrụ onye nlekọta ahuike? N’ịmaatụ, ọ bụ ndị gọvamentị ka ọ bụnke ndịnọrọ onwe ha?**
3. **Ọ bụ ebe a ka ịna-abịa karanata nlekọta ahuike?**
4. **Ruo ole oge ka ihe a ị na-akọ maka ya nọrọla?**
5. **Kedụ ka ndị dọkịnat na ndị nọọsụ si kpaso gị agwa?**
6. **Kedụihe ị chere maka ebe ahụ ị natara nlekọta ahuike?**
7. **Kedụ ihe ị ga-agbanwe ma ị chọọ ị gbanwe ihe ọbụla gbasara ihe ị gabigara, ewezuga ma ị mechara dị mma maọbụ na ị dịghị?**
8. **Ugbua, biko ndị ọzọ nille nọ n’otu, unu nwereike kpaa maka ihe ha chere gbasara akụkọ X.**
9. Nke a bụ nkebi nke ikpeazụ nke otu nlegara anya.

Nke a bụ agba nke ikpeazụ nke otu nlegara anya**. Ọtụtụn’ine unu akọla akụkọ maka ihe ha gabigara gbasara nlekọta ahuike, ma anyị chere ma o nwere onye n’ime ununọ n’ebe a onye darala n’ọrịa ma kpebie na ọ gaghị achọ nlekọta ahuike ọbụla. Ọbụrụna onwere onye n’ime unu gabigarala ụdị a, biko ịnwereike were oge kọọrọ anyị gbasara ya?**

1. Kpọọ mkpa hụ na nkọwa ha zara ajụjụ dịka achọrọ:

**Why did you not seek any health care?**

1. Onye ntụziaka ga-achọpụta ma detuo ndị otu anaghị etinye ọnụ n’ihe ana-eme. Ọbụrụna ọ na-ara ụfọdụ ahụ ikwulite okwu, webata sọtụ kaadị ma nye kaadị ntụzi nwere eserese gbasara ihe ụfọdụ dịka ego igosi na ego adịghị, nwoke igosi di, agadi nwaanyị igosi nne di, ihu ọdụ ahịa igosi azụmahịa, nye otu ọbụla ma jụọ ha ajụjụ ka kọwaa eserese nke dabara n’ihe mere ha ji chọọ maọbụ achọghị nlekọta ahuike.
2. Onye ntụziaka ga-ewete nkebi na mmechi site na i kele mmadụ niile maka nsonye ha n’otu nlegara anya:

**Akụkọ niile unu kọrọ taa dị nghomi ma dịkwa mma. Achọrọm ịkele unu nille maka isonyere anyị.**

**With seal of Dr. Ndubuisi Ahamefula**

**Professional member number 147 Nigerian Institute of Translators and Interpreters (NITI)**

**Lecturer, Department of Linguistics, Igbo & Other Nigerian Languages, University of Nigeria, Nsukka.**
