# Supplementary material for: “It’s what we perceive as different”: an interpretative phenomenological analysis of Nigerian women’s characterization of their health during the COVID-19 pandemic
Source: BMC Womens Health. 2024 Jul 18;24:409. doi: 10.1186/s12905-024-03259-w (PMC11256442; doi:10.1186/s12905-024-03259-w)
Supplement: Supplementary file 4 — Supplementary Material 4 [file 12905_2024_3259_MOESM4_ESM.pdf]

Usoro Igba ajụjụ ọnụ nhazitụ

Inyocha ihe ndị inyom na-agabiga n'inweta nlekọta ahụike n'oge ọnọdụ mgbasa ọrịa na Naijirịa: Nchọcha ke dabere na nkọwa isiokwu feminizm (gbasara mmenyere ndị nwaanyị)

Ndubata :

Ekele m gị maka ikweta ị nabata m taa. Tupu anyị amalite, maọbụrụ na ọ ga adị gị mma n'obi, ọ ga adị m mma ịnata gị ikikere ị rekọdụ ajụjụ ọnụ a. naanị ihe a ga eji rekọdụ a mee bụ sọọsọ idepụta ihe niile ekwuru n'ajụjụ ọnụ a iji hụna onweghi nkọwa ọbụla dị mkpa nke m hapụrụ idetu. Anam ekwe nkwa na mụ na onye nlekọta m na nchọcha a, na Mahadum nke Westeni naanị ga-abụ ndị ga-ahụ ihe dị na rekọkọdụ a, nke aga emebi ma afọ asaa gachaa. Ọ dịkwa gị n'aka ịhọrọ oge ọbụla ka ịgba ajụjụ ọnụ a na-aga n'ihu ị sị na ya achọchọghịzikwa ka e rekọdụ a ihe i kwuru. Ọzọ, matakwa na i nwereike ị họrọ ịjụ ịza ajụjụ ọbụla maọbụrụ nay a bụ ajụjụ adịchaghị gị mma ịza ya. N'ikpeazụ, achọrọ m ikwukwa ya ọzọ na I nwereike ị họrọ ịkwụsị ịgba ajụjụ ọnụ a ma sị ka ehichapụ ihe niile gbasara gị na nchọcha a oge ọbụla dị gị mma ka ajụjụ ọnụ na-aga n'ihu. Biko, kwenye na ị natala akwụkwọ ozi nkọwa nakwa nkwenye isonye n'ịgba ajụjụ ọnụ ma nabatakwa ka ka e rekọdụ a gị.

Ajụjụ gasị:

1. Kọọrọ m maka onwe gị.
   Mkpalite:
   a. Maka ego gị
   b. Maka ezinụlọ gị
   c. Maka agụmakwụkwọ gị

2. Biko I nwereike ịkọrọ m ihe ndị ụfọdụ ị gabigara mgbe ị nọ n'ọrịa ma chọọ nlekọta ahuike?
   Mkpalite:
   a. Kedụ onye ị ga-akpọtụrụ maọbụrụ na ị chọrọ enyemaka?
   b. Mgbe ị chọrọ inyeaka (kọha, keego, kemmetụta obi), kedụ ebe ị ga-aga?
   c. Ị chere na ndị ahụ nyere gị aka? N'ụzọ dị agha?
   d. O nwere onye ị na-achọkarị ịkpọtụrụ (di, nwanne nwaanyị, nne, onye agbataobi)? Kedụ ihe mere I ji a chọkarị ịkpọtụrụ ndị a kpọmkwem?
   e. Ọchịchọ ahụike gị ọ dị iche ugbu a (na mgbe) mgbasa ọrịa koro? Kọwaa?

3. I nwereike ị sị akparamagwa ọchịchị ahụike gị nyiri maọbụ dị mgbe ọrịa na-arịa nwa gị ma chọkwa nlekọta ahụike, tụmadụ kemgbe mgbasa ọrịa koro?
   a. Kedụ onye ị ga-akpọtụrụ maọbụrụ na ị chọrọ enyemaka?
   b. Mgbe ị chọrọ inyeaka (kọha, keego, kemmetụta obi), kedụ ebe ị ga-aga?
   c. Ị chere na ndị ahụ nyere gị aka? N'ụzọ dị agha?
   d. O nwere onye ị na-achọkarị ịkpọtụrụ (di, nwanne nwaanyị, nne, onye agbataobi)? Kedụ ihe mere I ji a chọkarị ịkpọtụrụ ndị a kpọmkwem?
   e. Ọchịchọ ahụike gị ọ dị iche ugbu a (na mgbe) mgbasa ọrịa koro? Kọwaa?

4. Biko I nwereike ịkọrọ m kedụ ka ọ dị bụ ị nata nlekọta ahuike dịka nwaanyịdị ime n'ụlọ ọgwụ n'oge mgbasa ọrịa koro?

   Mkpalite:

   a. Biko I nwereike ịkọrọ m maọbụrụna ị mụọ nwa n'oge mgbasa ọrịa koro? Nke ahụ ọbụ gịmbụịna-adị ime? Ma ọbụghị otu ahụ, ọ dị iche n'anya gị karịa mgbe ịmụrụ nwa nabụghị oge mgbasa ọrịa koro? Enwere ihe ndị siri ike? Kọọrọ m maka ya.
   b. Ma nkea abụghị ime mbụ gị, kedụ ụzọ ihe mgbu ịmụ nwa ị gabigara ji dị iche na ndị nke gara aga tupu oge mgbasa ọrịa koro?
   c. Ị natara nlekọta n'aka onye ọrụ nlekọta ahuike site n'ụlọ ahuike? Kedụ ụdị mmekọrịta gị na ndị ọrụ ụlọahuike nwere?
   d. Kedụ ka nlekọta ha si dị?
   e. O nyere aka ka ọ bụ na o nyeghị aka? (kpọọ mkpa hụna ịjụtara maka nkọwa nke ọma etu o si n ye aka/ihe mere o ji nye aka maọbụ otu o sighi nye aka) Ọ dị gị ka ndị ọrụ nlekọta ahuike o ghọtara gị nke ọma ma geeekwa gị ntị nke ọma gbasara mmetụta gị, uche gị na ochịchọ gị?
   f. Kedụ ihe mere I ji kpebie ị gawa ụlọ ọgwụ?
   g. Kedụ ihe I nwereike ịgbanwe?

5. Biko I nwereike ịkọrọ m maka oge ọbụla na ndụ okenye gị mgbe ị dara n'ọrịa ma kpebie nay a agaghị aga ụlọọgwụ?

   Mkpalite:

   a. Kedụ ihe mere I ji kpebie na ị gaghị aga?
   b. Kedụ ịihe ga-eme ka ọ dịrị mfe ịga ?
   c. Kedụ ihe i nwereike ịgbanwe?

6. Biko I nwereike ịkọwa otu n'ime ihe ndị ịmaakanihu gbasara ahuike ị gabigara n'oge mgbasa ọrịa koro?

   Mkpalite:

   a. I nwereike ị kọwa etu I si gabiga ya?
   b. I nwereike ị kwu maka atụmatụ ndị I ji merie ihe ndị ịmaakanihu ndịa niile?

7. Biko I nwereike ịkọrọ m maka mkpebi na nkwenye gị gbasara nlekọta ahuike?

   Mkpalite:

   a. Ị nwereike ịkọrọ m ihe ọpụtara gị inwe ahuike?
   b. Kedụ ka I si akpọ mkpa na I nwere ahụike, gị na ụmụaka gị?
   c. Kedụ ka mkpebi na nkwenye gị sirila gbanwe kemgbe mgbasa ọrịa koro?

8. Biko I nwereike ịkọrọ m mkpebi I mere gbasara ahụike gị n'ime ọnwa isii gara aga?
   a. I nwereike ị kọwara m ma ihe ndị ị gabigara o nwere mbelata kemgbe e wepụrụ mgbochi?
   b. Biko I nwereike ịkọrọ m ma mmekọrịta gị na ndị ọrụ ahuike ọdị mma karịa n'ime ọnwa isii gara aga?
