# Supplementary material for: “It’s what we perceive as different”: an interpretative phenomenological analysis of Nigerian women’s characterization of their health during the COVID-19 pandemic
Source: BMC Womens Health. 2024 Jul 18;24:409. doi: 10.1186/s12905-024-03259-w (PMC11256442; doi:10.1186/s12905-024-03259-w)
Supplement: Supplementary file 5 — Supplementary Material 5 [file 12905_2024_3259_MOESM5_ESM.docx]

**Jagorar Mai Gudanar da Tattaunawar Ƙungiya mai da hankali**

**Abubuwan da ake buƙata:**

• Rubutun sauti

**Ma'aikata:**

1. Malami na Farko (Dan takarar Ph.D.)

2. Mataimakin Bincike (Jhpeigo)

Ƙungiyar mai da hankali za ta kasance musamman ga matan da ke da yara 'yan ƙasa da shekaru 15 zuwa 49. A cikin kowace ƙungiyar mayar da hankali, kada a kasance fiye da mutane takwas. Masu gudanarwa za su gabatar da kansu kuma su bayyana dalilin da ya sa aka tattara rukunin. Yakamata su ba da taƙaitaccen bayanin yadda za a tafiyar da ƙungiyar mai da hankali. Bayanin ya kasance kamar haka:

**Barka da zuwa! Na gode don sanya hannu kan fom ɗin yarda da kuma komawa ga wannan taron. Muna haɗuwa don tattauna abubuwan da kuka samu lokacin neman kiwon lafiya tun lokacin bala'in. Abin da muka tattauna a nan zai kasance mai sirri. Zan fara da tambayar ku a taƙaice bayyana duk wata gogewa da kuka samu don neman lafiya. Zan kuma tambaye ku don bayyana wani gogewa da ba ku nemi kulawa ba da kuma dalilin da ya sa kuka zaɓi kin neman kulawa. Za mu tattauna wasu abubuwan da kuka samu dalla-dalla. Idan akwai tambayar da baku son amsawa, hakan yayi kyau. Da fatan za a raba abubuwan ku a fili. Babu amsa daidai ko kuskure.**

**Gabaɗayan zaman wataƙila zai ɗauki kimanin sa'o'i biyu. Za mu ba ku abin sha da kukis a wannan lokacin. Kamar yadda kuka ji lokacin da muka tattauna fom ɗin amincewa jiya, za mu yi rikodin zaman, amma babu abin da kuka faɗa a kaset ɗin da za a haɗa ku. Muna rikodin zaman ne saboda muna buƙatar samun cikakken bayani game da tattaunawar. Kuna iya zaɓar janyewa daga rukunin mayar da hankali a kowane lokaci. Koyaya, ku sani cewa ba zai yuwu a cire duk wani sharhi da kuka yi ba kafin fita. Ka tuna cewa ba dole ba ne ka amsa duk wata tambaya da za ta sa ka ji daɗi ko kuma ba ka son amsa. Sauran mahalarta ƙungiyar mayar da hankali sun san ainihin ku, kuma masu binciken ba za su iya ba da tabbacin cewa wasu a cikin wannan rukunin za su mutunta sirrin ƙungiyar ba. Aikin da muke yi a yau wani bangare ne na nazari da ake gudanarwa a jihohi 3: Ebonyi, Ogun, da Sokoto. Muna fatan gogewa da ra'ayoyin da kuke rabawa za su sanar da manufofi da tsare-tsaren kiwon lafiya na gaba ga mata da 'ya'yansu. Kafin mu fara, muna son ka sanya hannu a wata takardar yarda da ke tabbatar da cewa ka fahimci duk abin da muka bayyana kuma ka yarda da kiyaye duk bayanan da aka raba a wannan rukunin mayar da hankali.**

1. Sannan Malami ya fara tattaunawa da jumlar budewa kamar haka:

Yanzu, a taƙaice, wani zai iya bayyana halin da ake ciki lokacin da kuka tuntuɓi ma'aikacin lafiya? Wanene zai so farawa?

(Ba da izini ga mutane da yawa masu son yin magana don yin hakan a cikin lokacin da aka keɓe. Lokacin da ake tsammani: kamar mintuna 20)

2. Bayan kowa ya kammala labarinsa, sai ku nuna cewa kuna son ƙarin bayani kan wasu labarai dalla-dalla. Mai gudanarwa zai buƙaci zaɓar kusan benaye biyar don mai da hankali akai. Ya kamata a yi zaɓin labarun bisa ga ƙa'idodi masu zuwa: ya kamata labarun su kasance daban-daban dangane da nau'ikan sabis na kiwon lafiya da aka yi amfani da su da kuma yadda abubuwan suka kasance masu kyau ko mara kyau. Wannan ɓangaren rukunin mayar da hankali shine mafi tsayi kuma yakamata ya ɗauki kusan awa ɗaya.

**Labarun da kuka bayar suna da ban sha'awa sosai. Ina so a yanzu mu mayar da hankali kan cikakkun bayanai na kadan daga cikin wadannan labarai. Ina so in fara da labarin X. X, da fatan za a iya sake kwatanta abubuwan da kuka samu dalla-dalla. Yayin da X ke kwatanta gwaninta, Ina so kowa a cikin rukunin ya yi tunanin abin da ya faru da X da kuma yadda za su ji a yanayin X. Bayan X ya sake ba da labarin, zan yi wasu tambayoyi don ƙarin bayani. Ina so in buɗe tattaunawa don dukan ƙungiyar don yin tambayoyi da yin kowane sharhi game da yadda suka ji game da kwarewar X. Da zarar mun gama tattaunawa game da kwarewar X, za mu ci gaba da tattauna wani labari. Ina so mu kawo labarin hudu zuwa biyar a nan kamar haka?**

3. Bayan kammala labarin farko, mai gudanarwa ya kamata ya tuntuɓi wanda ake ƙara don ƙarin bayani ta hanyar amfani da tambayoyin kamar haka, idan ya cancanta kuma wanda aka ƙara bai rufe shi ba:

**a) Don Allah a ba ni ƙarin bayani game da wurin da kuka ga mai ba da lafiya? Misali, sabis na gwamnati ne ko na sirri?**

**b) Wannan shine wurin kulawa da kuka saba?**

**c) Tun yaushe ne lamarin da kuke bayyanawa?**

**d) Yaya likitoci/ma'aikatan jinya suka bi ku?**

**e) Me kuke tunani game da wurin da kuka sami kulawa?**

**f) Menene za ku canza idan kuna son canza wani abu game da gogewar, ban da ko kun sami lafiya ko a'a?**

**g) Yanzu, da fatan za a so sauran ƙungiyar su tattauna yadda suka ɗauki labarin X.**

4. Wannan shine sashe na ƙarshe na ƙungiyar mai da hankali.

**Wannan shine bangare na karshe na rukunin mayar da hankalinmu. Yawancinku sun ba da labari game da abubuwan da suka shafi kiwon lafiya, amma mun yi mamakin ko ɗayanku a nan ya yi rashin lafiya amma kuma ya zaɓi kada ya nemi kulawar lafiya. Idan ɗayanku ya sami wannan ƙwarewar, da fatan za ku ba da lokaci kuna gaya mana game da shi?**

5. Tabbatar da bayanin su ya amsa tambayar:

**Me ya sa ba ku nemi lafiya ba?**

6. Malami ya lura da kasancewar mahalarta marasa aiki. Idan wasu suna samun wahalar yin magana, gabatar da nau'in katin kuma a ba da katunan alamar da aka riga aka buga tare da hotunan batutuwa kamar kuɗi don rashin kuɗi, namiji don ma'aurata, tsofaffin mata don surukai, kantin sayar da kayayyaki, ga kowace ƙungiya kuma ka tambaye su don gano ko wane hoto ya bayyana dalilin da ya sa suka zaɓa don ko ba su nemi kulawa ba.

7. Malami ya rufe taron da godiya ga kowa da kowa bisa yadda ya shiga cikin rukunin:

**Labaran ku sun kasance masu fa'ida da ban sha'awa. Ina so in gode muku duka don halartarku.**

**With the seal of Dr. Ndubuisi Ahamefula**

**Professional member number 147 Nigerian Institute of Translators and Interpreters (NITI)**

**Lecturer, Department of Linguistics, Igbo & Other Nigerian Languages, University of Nigeria, Nsukka.**

ndubuisi.ahamefula@unn.edu.ng
