# Supplementary material for: “It’s what we perceive as different”: an interpretative phenomenological analysis of Nigerian women’s characterization of their health during the COVID-19 pandemic
Source: BMC Womens Health. 2024 Jul 18;24:409. doi: 10.1186/s12905-024-03259-w (PMC11256442; doi:10.1186/s12905-024-03259-w)
Supplement: Supplementary file 6 — Supplementary Material 6 [file 12905_2024_3259_MOESM6_ESM.pdf]

Jagorar Tambayoyi Mai Tsari-tsare

Binciko abubuwan da mata ke samun kulawa yayin bala'i a Najeriya:

Nazari mai inganci na Feminist.

Preamble:

Na gode da yarda da saduwa da ni a yau. Kafin mu fara, idan ba ku damu ba, Ina son izinin ku don yin rikodin wannan hirar. Za a yi amfani da rikodin ne kawai don rubuta hirar da kuma tabbatar da cewa ban rasa kowane muhimmin bayani ba. Ina ba da tabbacin cewa ni, mai kula da ni, da Jami'ar Yammacin Turai ne kawai za mu iya samun damar yin rikodin, wanda za a lalata bayan shekaru bakwai. Kuna iya zaɓar ƙin yin rikodin a kowane lokaci a cikin tsarin tambayoyin. Hakanan, lura cewa zaku iya zaɓar ƙin amsa kowace tambaya idan ba ku ji daɗin amsawa ba. A ƙarshe, ina so in sake maimaita cewa za ku iya zaɓar dakatar da hirar kuma ku nemi a share bayananku daga binciken a kowane lokaci yayin hirar. Da fatan za a tabbatar da cewa kun karɓi wasiƙar bayani da yarda don yin hira da yin rikodin.

Tambayoyi:

1. Bani labarin kanku.

Bincike:

a. Bayanan kudi

b. Asalin dangi

c. Asalin ilimi

2. Za ku iya gaya mani game da wasu abubuwan da kuka fuskanta lokacin da kuke rashin lafiya kuma kuna buƙatar kulawa?

Bincike:

a. Wanene za ku tuntuɓi idan kuna buƙatar taimako?

b. Lokacin da kuke buƙatar taimako (zamantakewa, tattalin arziki, motsin rai), ina za ku?

c. Kuna tsammanin waɗannan mutanen sun taimaka? Ta wace hanya ce?

d. Shin akwai wanda kuke son tuntuɓar (miji, 'yar'uwa, uwa, maƙwabci)? Me yasa kuke tuntubar su musamman?

e. Shin (ko) neman lafiyar ku ya bambanta yayin bala'in COVID-19? Ta yaya haka?

3. Za ku iya cewa halin ku na neman lafiya iri ɗaya ne ko kuma ya bambanta lokacin da yaronku ba shi da lafiya kuma yana buƙatar kulawa, musamman tun bayan cutar ta COVID-19?

a. Wanene za ku tuntuɓi idan kuna buƙatar taimako?

b. Lokacin da kuke buƙatar taimako (zamantakewa, tattalin arziki, motsin rai), ina za ku?

c. Kuna tsammanin waɗannan mutanen sun taimaka? Ta wace hanya ce?

d. Shin akwai wanda kuke son tuntuɓar (miji, 'yar'uwa, uwa, maƙwabci)? Me yasa kuke tuntubar su musamman?

e. Shin (ko) neman lafiyar ku ga yaranku ya bambanta yayin bala'in COVID-19? Ta yaya haka?

4. Za a iya gaya mani yadda ake samun kulawa a matsayin uwa mai jiran gado a asibiti yayin bala'in COVID-19?

Bincike:

a. Za a iya gaya mani idan kun haihu a lokacin wannan cutar ta COVID-19? Wannan shine cikin ku na farko? Idan ba haka ba, ya bambanta da lokacin da kuka haihu a wajen cutar ta COVID-19? Akwai wasu matsaloli? Ku gaya mani game da shi.

b. Idan ba cikin ku na farko ba, ta waɗanne hanyoyi ne ƙwarewar aikinku ta bambanta da waɗanda suka kasance kafin cutar ta COVID-19?

c. Shin kun sami kulawa daga ma'aikacin kiwon lafiya? Yaya yanayin hulɗarku da ƙwararrun kiwon lafiya ya kasance?

d. Yaya kulawa?

e. Menene taimako ko bai taimaka ba? (tabbatar da neman ƙarin bayani kan yadda/me yasa taimako/marasa taimako)Shin kun ji fahimtar ku da kuma sauraron ku daga ma'aikatan kiwon lafiya daban-daban game da ji, tunani, da buƙatun ku?

f. Me ya sa ka zabi ka je asibiti?

g. Me zaku canza.

5. Za a iya gaya mani wani lokaci a rayuwarka ta balaga da ka yi rashin lafiya amma ba za ka je asibiti ba?

Bincike:

a. Me ya sa ka zabi ba za ka je ba?

b. Menene zai sauƙaƙa muku tafiya?

c. Me zaku canza?

6. Shin za ku iya kwatanta ɗaya daga cikin abubuwan da kuka fuskanta na lafiya mafi ƙalubale yayin wannan cutar ta COVID-19?

Bincike:

a. Za ku iya bayyana yadda kuka yi da shi?

b. Za ku iya magana game da dabarun da kuka yi amfani da su don magance waɗannan ƙalubalen?

7. Za ku iya gaya mani game da dabi'unku da imaninku game da kiwon lafiya?

Bincike:

a. Za a iya gaya mani abin da ake nufi da zama lafiya a gare ku?

b. Ta yaya kuke kula da lafiyar kanku da yaranku?

c. Ta yaya dabi'unku da imaninku suka canza tun bayan cutar ta COVID-19?

8. Za a iya gaya mani game da shawarar lafiyar ku a cikin watanni 6 da suka gabata?

a. Za ku iya yin bayani idan abubuwanku sun inganta tun lokacin da aka ɗaga hani?

b. Za a iya gaya mani idan dangantakar ku da mai kula da lafiyar ku ta inganta a cikin watanni shida da suka gabata?

With the seal of Dr. Ndubuisi Ahamefula

Professional member number 147 Nigerian Institute of Translators and Interpreters (NITI)

Lecturer, Department of Linguistics, Igbo & Other Nigerian Languages, University of Nigeria, Nsukka.

ndubuisi.ahamefula@unn.edu.ng
