# Supplementary material for: “It’s what we perceive as different”: an interpretative phenomenological analysis of Nigerian women’s characterization of their health during the COVID-19 pandemic
Source: BMC Womens Health. 2024 Jul 18;24:409. doi: 10.1186/s12905-024-03259-w (PMC11256442; doi:10.1186/s12905-024-03259-w)
Supplement: Supplementary file 7 — Supplementary Material 7 [file 12905_2024_3259_MOESM7_ESM.pdf]

Focus Group Discussion Facilitator Guide

Required resources:
- Audio records

Staff:
1. Primary facilitator (Ph.D. Candidate)
2. Research assistants (Jhpeigo)

The focus group will be specific to women with under-five children aged 15 to 49 years old. Within each focus group, there should be no more than eight people. The facilitators will introduce themselves and explain why the focus group has been gathered. They should give a brief outline of how the focus group will be run. The explanation should read as follows:

> **Welcome! Thank you for signing the consent forms and for returning to this meeting. We are meeting to discuss your experiences when seeking health care since the pandemic. What we discuss here will remain confidential. I will start by asking you to briefly describe any experience you have had seeking health care. I will also ask you to describe an experience you had not sought care and why you chose not to seek care. We will then discuss some of your experiences in more detail. If there is a question you do not want to answer, that is ok. Please share your experiences openly. There are no right or wrong answers.**
> **The entire session will probably take about two hours. We will provide you with something to drink and cookies during this time. As you heard when we discussed the consent form yesterday, we will record the sessions, but nothing you say on tape will be linked to you. We are recording the sessions because we need to have an accurate account of the discussions. You can choose to withdraw from the focus group at any time. However, know that it will be impossible to remove any comments that you may have made before exiting. Remember that you do not have to answer any questions that make you uncomfortable or that you do not want to answer. Your identity is known by other focus group participants, and the researchers cannot guarantee that others in this group will respect the confidentiality of the group. The work we are doing today is part of a study that is being conducted in 3 states: Ebonyi, Ogun, and Sokoto States. We hope that the experiences and perspectives that you share will inform future policies and health plans for women and their children. Before we begin, we would like you to sign another consent form confirming that you understand everything we explained and that you agree to keep all information shared at this focus group confidential.**

1. The facilitator then starts the discussion with the following opening sentence:
   Now, very briefly, would someone please describe a situation when you consulted a health care provider? Who would like to start?
   (Allow as many individuals as possible willing to speak to do so within the allocated time. Expected time: about 20 minutes)

2. After everyone has completed their stories, indicate that you would like to focus on a few more stories in more detail. The moderator will need to select about five stories to focus on. The selection of stories should be made according to the following principles: the stories should be diverse concerning the different types of health services used and how good or bad the experiences were. This part of the focus group is the longest part and should take about an hour.
    The stories that you have told are very interesting. I would now like us to focus on the details of a few of these stories. I want to start with X's story. X, could you please describe your experience again in a little more detail. While X is describing the experience, I would like everyone else in the group to think about what happened to X and how they would have felt in X's situation. After X has retold the story, I will ask some questions for clarification. I would then like to open the discussion for the whole group to ask questions and make any comments on their reaction to X's experience. Once we have finished discussing X's experience, we will go on to discuss another story. I would like us to cover four to five of the stories told here today in a similar way. X, would you please start?

3. After completion of the first story, the moderator should prompt the respondent for more information using the following questions, if necessary and not covered by the respondent:
    a) **Please tell me more about the place where you saw the health care provider? For example, was it a government or private service?**
    b) **Was this your usual place of care?**
    c) **How long ago was the incident you are describing?**
    d) **How were you treated by the doctors/nurses?**
    e) **What did you think of the place where you received care?**
    f) **What would you change if you wanted to change anything about the experience, apart from whether you got better or not?**
    g) **Now, please would the rest of the group like to discuss their reaction to X's story.**

4. This is the final section of the focus group.

This is the final part of our focus group. Many of you have told stories about health care experiences, but we wondered whether any of you here have been ill but then chose not to seek any health care. If any of you have had that experience, would you please spend time telling us about it?

5. Make sure their description answers the question:
    Why did you not seek any health care?

6. The facilitator should note the presence of inactive participants. If some are finding it difficult to speak up, introduce the card sort and provide preprinted cue cards with images of issues such as money for lack of finance, man for a spouse, older women for mother-in-law, a storefront for business, to each group and ask them to identify which images describe why they choose to or not seek care.
7. The facilitator should close the session by thanking everyone for their participation in the focus group:
    Your stories have been very insightful and interesting. I want to thank you all for your participation.
