# Supplementary material for: “It’s what we perceive as different”: an interpretative phenomenological analysis of Nigerian women’s characterization of their health during the COVID-19 pandemic
Source: BMC Womens Health. 2024 Jul 18;24:409. doi: 10.1186/s12905-024-03259-w (PMC11256442; doi:10.1186/s12905-024-03259-w)
Supplement: Supplementary file 8 — Supplementary Material 8 [file 12905_2024_3259_MOESM8_ESM.pdf]

Semi-structured Interview guide

Exploring the experiences of women accessing care during a pandemic in Nigeria: A Qualitative Feminist Phenomenological Study

Preamble:

Thank you for agreeing to meet with me today. Before we begin, if you do not mind, I would like your permission to record this interview. The recording will only be used to transcribe the interview and ensure that I do not miss any vital information. I guarantee that I, my supervisor, and the University of Western will be the only ones with access to the recording, which will be destroyed after seven years. You may choose to decline to be recorded at any point in the interview process. Also, note that you can choose to refuse to answer any questions if you find them uncomfortable answering. Finally, I want to reiterate that you can choose to stop the interview and request that your data be deleted from the study at any point during the interview. Please confirm that you have received the letter of information and consent to be interviewed and recorded.

Questions:

1. Tell me about yourself.
   Probes:
   a. Financial background
   b. Family background
   c. Educational background

2. Could you tell me about some of your experiences when you are ill and need care?
   Probes:
   a. Who would you contact if you need assistance?
   b. When you need help (social, economic, emotional), where do you go?
   c. Do you think those people were helpful? In what ways?
   d. Is there anyone you tend to consult (husband, sister, mother, neighbour)? Why do you consult them in particular?
   e. Is (or has) your health-seeking been different during the COVID-19 pandemic? How so?

3. Would you say your health-seeking behaviour is similar or different when your child is ill and needs care, especially since the COVID-19 pandemic?
   a. Who would you contact if you need assistance?
   b. When you need help (social, economic, emotional), where do you go?
   c. Do you think those people were helpful? In what ways?
   d. Is there anyone you tend to consult (husband, sister, mother, neighbour)? Why do you consult them in particular?
   e. Is (or has) your health-seeking for your child(s) been different during the COVID-19 pandemic? How so?

4. Could you tell me what it is like to receive care as an expecting mother at the hospital during the COVID-19 pandemic?

   Probes:

   a. Could you tell me if you gave birth during this COVID-19 pandemic? Was this your first pregnancy? If not, did it seem different than when you had given birth outside the COVID-19 pandemic? Were there any difficulties? Tell me about it.
   b. If not your first pregnancy, in what ways was your labour experience different from the ones before the COVID-19 pandemic?
   c. Did you receive care from a health care provider? What was the nature of your interaction with health care professionals?
   d. What was the care like?
   e. What was helpful or not helpful? (make sure to ask for elaboration on how/why helpful/unhelpful) Did you feel understood and listened to by different healthcare providers regarding your feelings, thoughts, and needs?
   f. Why did you choose to go to the hospital?
   g. What would you change?

5. Could you tell me of any times in your adult life you were ill but chose not to go to the hospital?

   Probes:

   a. Why did you choose not to go?
   b. What would have made it easy for you to go?
   c. What would you change?

6. Could you describe one of your most challenging health experiences during this COVID-19 pandemic?

   Probes:

   a. Can you explain how you dealt with it?
   b. Can you talk about the strategies that you used to deal with these challenges?

7. Could you tell me about your values and beliefs about health care?

   Probes:

   a. Can you tell me what it means to be healthy to you?
   b. How do you maintain good health for yourself and your children?
   c. How have your values and beliefs changed since the COVID-19 pandemic?

8. Could you tell me about your health decision in the last 6 months?
   a. Can you explain if your experiences have improved since the lifting of restrictions?
   b. Can you tell me if your relationship with your health provider has improved in the last six months?
